# Monitoring the Evolution of SARA Fractions During Asphalt Binder Aging by Automated HPLC and Correlation with FTIR Oxidation Indices

**DOI:** 10.3390/ma19153249

**Published:** 2026-08-01

**Authors:** Giovanni Polacco, Chiara Riccardi, Pietro Leandri, Massimo Losa, Sara Filippi

**Affiliations:** Department of Civil and Industrial Engineering, University of Pisa, Largo Lucio Lazzarino 1, 56122 Pisa, Italy; chiara.riccardi@unipi.it (C.R.); pietro.leandri@unipi.it (P.L.); massimo.losa@unipi.it (M.L.)

**Keywords:** asphalt binder aging, SARA fractions, automated HPLC, FTIR oxidation indices, colloidal instability index

## Abstract

**Highlights:**

- Automated HPLC-SARA analysis was applied to monitor thermo-oxidative aging of asphalt binders.
- Aromatics progressively decreased while asphaltenes increased with aging.
- Colloidal instability increased consistently with progressive oxidation.
- The colloidal instability index showed a strong correlation with FTIR carbonyl oxidation markers.
- Automated HPLC-SARA provided repeatable compositional indicators of asphalt aging.

**Abstract:**

The qualitative evolution of SARA fractions during asphalt binder aging is well established, but further validation is needed to demonstrate whether automated HPLC-SARA analysis can provide repeatable compositional indicators for systematic aging studies. In this work, the applicability of a previously optimized automated high-performance liquid chromatography (HPLC) workflow for saturate, aromatic, resin, and asphaltene (SARA) fractionation was evaluated for the monitoring of thermo-oxidative aging. Four penetration-grade asphalt binders were subjected to short-term aging and multiple long-term aging cycles, and the resulting SARA distributions were used to calculate the colloidal instability index (*I_c_*). Fourier-transform infrared spectroscopy (FTIR) was used as an independent reference technique to evaluate carbonyl and sulfoxide oxidation indices. The automated HPLC-SARA method provided repeatable compositional indicators across all asphalt binders and aging conditions. As expected, aromatics progressively decreased whereas asphaltenes increased, while saturates showed only limited variations and resins behaved as an intermediate operational fraction. More importantly, *I_c_* increased consistently with aging and showed a strong correlation with the FTIR carbonyl index (R^2^ = 0.84 when all asphalt binders and aging conditions were considered), whereas the sulfoxide index showed a more asphalt binder-dependent response. These results demonstrate that automated HPLC-SARA analysis can provide aging-sensitive compositional and colloidal indicators that are chemically consistent with independent FTIR oxidation markers. The proposed workflow represents a complementary tool for asphalt binder aging research and may support future studies aimed at linking compositional evolution with rheological and durability-related properties.

## 1. Introduction

Asphalt binders are subjected to environmental stresses that induce progressive alterations in their physical integrity and chemical composition, with consequent degradation of their performance. Mechanical stresses due to traffic loads and periodic temperature variations, as well as chemical reactions induced by reactive oxygen species in combination with moisture and ultraviolet radiation, contribute to this phenomenon, which is generally referred to as aging. The first appreciable alteration of the asphalt binder properties, which already occurs during paving, is usually described as short-term aging. In contrast, the changes that occur slowly during the in-service life can be monitored and appreciated only over a period of years and are referred to as long-term aging. Asphalt aging has been extensively studied in recent decades, and an abundant literature is available on this subject. Among recent contributions, several review papers address different aspects of aging. First, to simulate and predict the aging behaviour of asphalt binders, it is essential to reproduce it at the laboratory scale. This has led to the development of several artificial aging procedures, either included in standards or not [1,2,3,4]. Aging can be investigated at different scales, ranging from macroscopic approaches based on rheological and thermal characterization to mesoscopic compositional analyses, microscopic structural observations, and molecular-level techniques such as gel permeation chromatography and molecular simulations [5,6,7]. Monitoring one or more of these properties is useful for defining performance indicators that quantify in-field aging or determine end-of-life criteria [8]. Another widely investigated aspect of aging concerns strategies to delay it, for example through the use of antioxidants [9] or rejuvenating additives to improve the recycling of end-of-life pavements [10].

Since aging is directly related to changes in asphalt composition, the latter has been widely investigated, mainly through Fourier-transform infrared analysis (FTIR), which has demonstrated that the main functional groups formed during oxidative aging are ketones, anhydrides, carboxylic acids and sulfoxides [11,12]. A quantitative analysis of such groups has been suggested as a reliable tool for defining aging parameters, such as the carbonyl and sulfoxide indices, based on C=O and S=O groups respectively [13,14]. As the formation of the above-mentioned functional groups alters the composition and polarity of many asphalt molecules, aging may also be monitored through the so-called SARA fractions. These fractions derive from the pioneering works of Corbett that chromatographically subdivide asphalt components into four groups of increasing polarity, namely saturates, aromatics, resins and asphaltenes [15]. The relative quantities of these fractions determine the colloidal equilibrium and the overall macroscopic properties of the asphalt binder. Therefore, the evolution of SARA fractions can provide an operational indicator of asphalt binder aging, reflecting changes in polarity, solubility class, and colloidal balance. Indeed, the colloidal instability index (*I_c_*), or its inverse, the colloidal stability index (*CI*), can be used as an indicator of the aging level.

$$I_{c}=\frac{1}{CI}=\frac{saturates+asphaltenes}{aromatics+resins}$$

A low value of *I_c_* (high *CI*) indicates the ability of the lighter oily fractions to disperse asphaltene micelles in a stable colloidal structure. The reduction of aromatics and the increase in asphaltenes, typically associated with oxidative aging, lead to an increase in colloidal instability (i.e., a decrease in stability). In the present study, *I_c_* was calculated from the SARA distribution obtained by the automated HPLC method; therefore, its absolute value is not intended to be directly interchangeable with *I_c_* values calculated from other SARA protocols. It should also be emphasized that SARA fractions are operational solubility-based classes. Therefore, their evolution during aging should not be interpreted solely as a direct measure of oxygen uptake or as a simple consequence of the formation of oxygen-containing functional groups. In addition to oxidation, changes in apparent molecular size, aromaticity, hydrogen content, molecular association, and solubility class may contribute to the redistribution of material toward more resin- or asphaltene-like fractions [16,17,18,19,20].

Based on ASTM D4124 [21], which is commonly used for asphalt binders, the four SARA fractions are obtained through a preliminary precipitation of asphaltenes in n-heptane (nC7), followed by chromatographic separation of the remaining maltenes in an open column filled with activated alumina. However, SARA fractionation is not uniquely defined even within standardized procedures. For example, ASTM D2007 [22] also describes the separation of saturates, aromatics, resins, and asphaltenes, but uses n-pentane rather than nC7 as the precipitating solvent. Since the separation between asphaltenes and maltenes is governed by solubility in the selected paraffinic solvent, changing the solvent inevitably affects the operational boundary between these fractions and therefore leads to different SARA distributions. This further confirms that SARA fractions are operationally defined, and that derived parameters such as the colloidal instability index, should always be interpreted with reference to the specific analytical procedure used.

Conventional open-column SARA procedures are tedious, non-automated, do not guarantee good reproducibility, and require long analysis times as well as significant amounts of high-purity solvents. For these reasons, alternative procedures based on either thin-layer chromatography–flame ionization detection (TLC-FID) or high-performance liquid chromatography (HPLC) have been developed. TLC-FID has the advantage that a specifically designed commercial instrument, known as Iatroscan^®^, is available and can be used with the same procedure irrespective of the research group [7,23,24,25,26,27,28]. This is probably the main reason why most of the literature on chromatographic separation of SARA fractions is based on TLC-FID [7,25,29,30,31,32,33,34]. However, TLC-FID is time-consuming and has several drawbacks including strong operator dependence, limited reproducibility, and the low flammability of some asphalt components [35]. In contrast, no HPLC system specifically designed for SARA fractionation is commercially available, and several different configurations and procedures have been developed over the last decades. This has led to continuous improvements in the method, ultimately enabling fully automated systems with fast and reproducible results [36,37,38]. This makes HPLC a particularly suitable approach when many samples need to be analysed. Nevertheless, since no single commercial instrument or universally accepted procedure exists, interlaboratory reproducibility may remain an issue. In other words, the results are internally consistent but not directly comparable with those obtained using different HPLC configurations or other chromatographic methods.

Among non-chromatographic analytical techniques, the use of FTIR was also suggested for SARA analysis. However, FTIR identifies functional groups that can be present in more than one of the four fractions. Therefore, this is an indirect measurement that needs to be correlated with the relative amounts of the SARA fractions, which is a very challenging task in chemically complex systems such as asphalt. Weigel and Stephan derived the relative content of maltenes and asphaltenes from the FTIR spectra [39], but concluded that the structural similarity between aromatics and resins makes it very difficult to quantify the maltenes subfractions. In contrast, Li et al. developed an algorithm to obtain the four SARA fractions directly from the infrared spectra [40]. The method was calibrated by comparing FTIR and TLC-FID data from 21 asphalt binders subjected to four different degrees of aging. The results were promising; however, the 21 asphalt binders were obtained by mixing only two base asphalts in varying proportions, thus creating a very homogeneous and self-consistent dataset. This raises concerns about the exportability of the proposed algorithm.

As already mentioned, previous HPLC-SARA studies have contributed substantially to the development and optimization of faster chromatographic approaches for group-type fractionation. These works have mainly addressed analytical aspects, such as chromatographic configuration, detector response, fraction separation, method repeatability, or comparison with conventional SARA procedures. However, limited attention has been paid to whether automated HPLC-SARA can be systematically applied to follow progressive asphalt binder aging and to generate compositional indicators consistent with independent oxidation markers. In our previous work, the automated HPLC-SARA workflow used here was described and compared with ASTM D4124, clarifying several critical methodological aspects of the procedure [37]. That comparison showed that the two methods provide internally consistent but not directly interchangeable SARA distributions, because the resulting fractions depend on the specific separation mechanism, solvent conditions, chromatographic supports, and operational fraction boundaries. For this reason, the automated HPLC-SARA workflow is used here as a self-consistent analytical framework to monitor relative compositional changes during aging, rather than as a direct replacement of ASTM D4124 on an absolute fraction-by-fraction basis. Building on the previous methodological assessment, the workflow is applied to thermo-oxidative aging, while FTIR-derived oxidation indices are used as an independent reference to evaluate the chemical consistency of the HPLC-derived colloidal instability index.

Previous studies already investigated the evolution of oxidation products within individual SARA fractions using FTIR, providing valuable information on the chemical pathways involved in asphalt binder aging [11]. However, although FTIR-based oxidation indices such as the carbonyl index (*I_C=O_*) and the sulfoxide index (*I_S=O_*) are widely used to monitor chemical aging in asphalt binders [12,13,14,41], direct quantitative relationships between these indices and colloidal parameters derived from SARA analysis, such as *I_c_*, remain largely unexplored. The present study addresses this gap by systematically comparing the evolution of *I_c_* obtained through automated HPLC-SARA analysis with FTIR oxidation indices for four penetration-grade asphalt binders subjected to progressive thermo-oxidative aging. The aim is not to rediscover the well-established qualitative evolution of SARA fractions during aging, but to demonstrate the applicability of the previously optimized automated HPLC-SARA workflow as a reliable tool for monitoring asphalt binder aging. In particular, the study evaluates whether the method can consistently track aging-induced compositional changes over multiple asphalt binders and aging conditions, and whether the resulting colloidal instability index is coherent with oxidation markers obtained from FTIR, a completely independent and widely used technique for asphalt aging assessment. In this framework, FTIR is used not as an alternative method for SARA quantification, but as an independent chemical reference to verify whether the compositional evolution captured by HPLC-SARA follows the same aging progression indicated by the formation of oxygen-containing functional groups. Therefore, the contribution of this work lies in demonstrating that automated HPLC-SARA analysis can provide repeatable aging-sensitive compositional indicators and can be effectively combined with complementary techniques for a more comprehensive assessment of asphalt binder aging.

## 2. Materials and Methods

### 2.1. Materials

nC7, dichloromethane (DCM), and methanol (MeOH) were of HPLC grade, purchased from Merck Life Science (Darmstadt, Germany), and used as received. Four conventional paving-grade asphalt binders, classified according to penetration grade as 35–50, 50–70, 70–100, and 160–220, were investigated. These binders were selected to cover a broad range of consistencies, from hard to soft binders, and to evaluate whether the automated HPLC-SARA workflow provides consistent aging-sensitive indicators across different initial compositions. The main physical properties of the unaged binders are summarized in Table 1. Each binder was analysed in the unaged state and after the artificial aging treatments described below.

### 2.2. Artificial Aging Procedure

The asphalt binders were subjected to thermo-oxidative aging using the Rolling Thin Film Oven Test (RTFO) for short-term aging, followed by Pressure Aging Vessel (PAV) treatment for long-term aging. RTFO aging was performed according to ASTM D2872 [42] (Standard Test Method for Effect of Heat and Air on a Moving Film of Asphalt Binder), with samples maintained in rotating flasks for 85 min at 163 °C under an air flow of 4000 mL/min. PAV aging was conducted according to ASTM D6521 [43] (Standard Practice for Accelerated Aging of Asphalt Binder Using a Pressurized Aging Vessel), with static samples maintained at 110 °C under air at a pressure of 2.1 MPa, for 20 h.

To extend the investigated aging range beyond conventional laboratory aging and facilitate the evaluation of progressive compositional changes, one or two additional PAV cycles were applied, corresponding to total PAV aging times of 40 and 60 h, respectively. These extended PAV treatments were not intended to reproduce specific field aging times or to establish a direct equivalence with in-service pavement aging. Rather, they were used as accelerated laboratory aging conditions to generate a broader set of oxidation states under controlled conditions, allowing the ability of the automated HPLC-SARA workflow to track progressive compositional and colloidal changes to be assessed over an extended aging sequence. Throughout the manuscript, the asphalt binders are referred to as unaged, RTFO, PAV1, PAV2, and PAV3 according to the applied aging level. It should be noted that RTFO aging, and potentially extended PAV aging, may also involve the loss of volatile or low-molecular-weight components. In the present study, mass loss during aging was not quantified separately, because the objective was to monitor relative compositional changes by HPLC-SARA rather than to establish a complete mass balance during aging. Moreover, even if total mass loss had been measured, its attribution to a specific SARA fraction would not have been straightforward, since volatile or low-molecular-weight components may be present in different operational chromatographic classes rather than being limited to the saturate fraction. Therefore, the reported SARA evolution should be interpreted as the result of combined aging-induced processes, including oxidation, changes in solubility class, molecular association, relative redistribution among fractions, and possible volatilization of lighter components.

### 2.3. Automated HPLC-SARA Analysis

**Figure 1:**
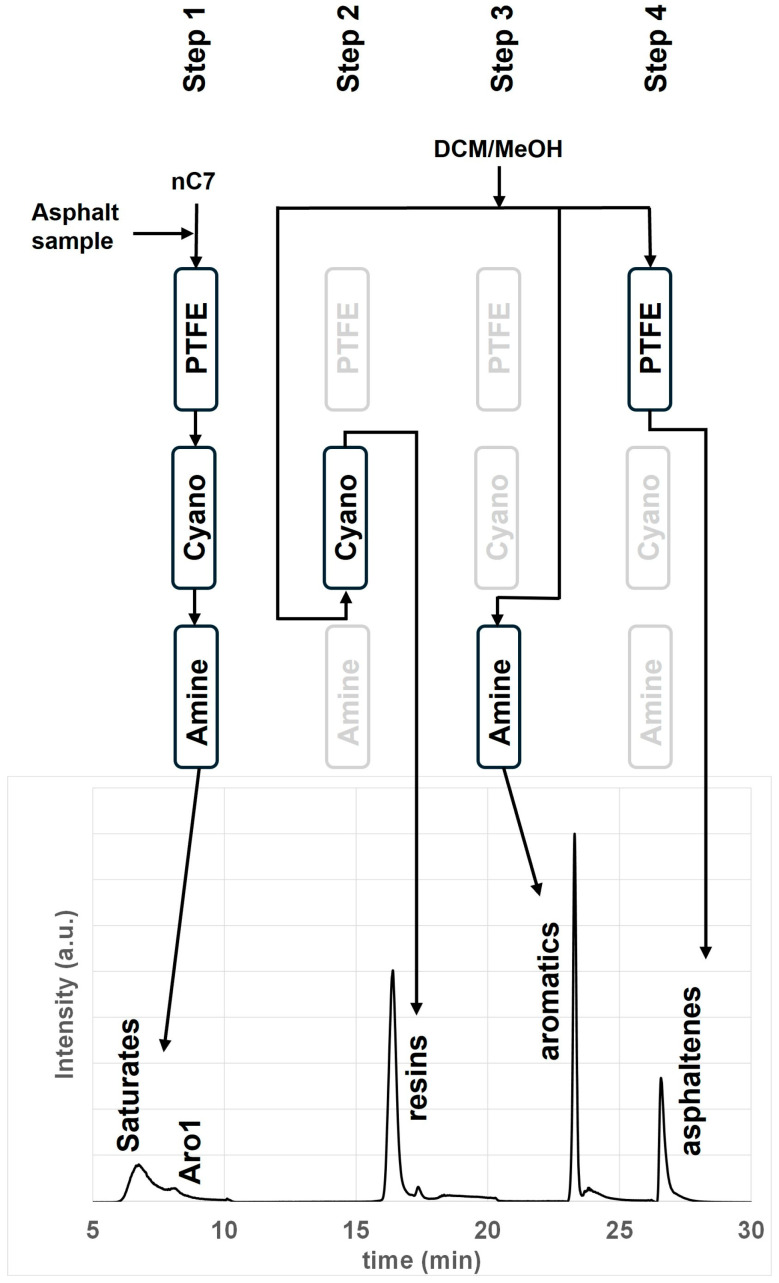
Schematic representation of HPLC procedure and corresponding chromatogram recorded for unaged asphalt 50–70. In step 2 the cyano column is eluted in backflush.

The automated HPLC-SARA procedure can be divided into four main steps: sample preparation, selective retention of fractions on the column sequence, automated solvent/valve switching for fraction elution, and chromatogram integration. In the first step, the asphalt binder solution (10 μL with a concentration of 4 mg/mL corresponding to 40 μg of asphalt binder) is injected under nC7 flow, which induces asphaltene precipitation and retention on the PTFE column. The remaining maltenic components then pass through the cyano and amino columns, where resins and most aromatic compounds are selectively retained, while saturates and light aromatic compounds elute first and reach the detector. In the subsequent steps, automated valve switching and solvent change to DCM/MeOH allow the retained asphaltenes, resins, and aromatics to be eluted separately and quantified from the corresponding chromatographic peaks. The HPLC system was a Jasco instrument equipped with a Jasco AS4050 autosampler, a Jasco BS-4000-1 bottle stand, a Jasco PU-4180 quaternary gradient pump, five 6-position Jasco HV-4380 switching valves, a Jasco CO-4065 column oven set at 40 °C (JASCO Corporation, Hachioji, Japan), and a SEDEX 85 LT-ELS evaporative light scattering (ELS) detector (SEDERE) (SEDERE SAS, Olivet, France), set at 35 °C and 3.3 bar nitrogen flow. Three columns were used: a 10 × 7.8 mm stainless steel column filled with PTFE powder (average particle size 675 mm from Merck Life Science) and two chromatographic columns—a cyano-bonded silica gel column (HyperClone 5 mm CN 120 Å, 250 × 4.6 mm), and an aminopropyl bonded silica gel column (Luna 5 mm Silica 100 Å, 250 × 4.6 mm)—both supplied by Phenomenex. The chromatographic configuration was designed to achieve sequential isolation of asphaltenes, resins, and aromatic fractions under fully automated operation. Both the apparatus and the automated procedure were described in detail in previous work [37]. Only the main operating conditions are summarized here for completeness. In brief, approximately 1 g of asphalt was dissolved in 40 mL of DCM at room temperature. The solution was gently stirred for a few minutes and then left overnight to ensure complete dissolution of all asphalt components. A small portion of the solution was further diluted in DCM to obtain a concentration of 4 mg/mL. A volume of 10 mL of the solution was injected into the HPLC system and carried by a 1.0 mL/min flow of nC7 through the PTFE, cyano and amino columns arranged in series. Since nC7 induces their precipitation, asphaltenes are immediately captured by the PTFE column. The remaining components pass through the cyano column, where resins are retained, and subsequently through the amino column, which retains most of the aromatic fraction. The remaining aromatics (referred to as Aro1) and saturates elute from the last column and reach the detector. Aro1 compounds are assumed to contain a single aromatic ring [44]; however, the presence of saturated substituents gives them chromatographic behavior similar to that of saturates [45]. Following this preliminary phase, the mobile phase is changed to DCM/MeOH (98/2 *v*/*v*), and the valves are automatically adjusted to separately recover the asphaltenes, resins and remaining aromatics from the three columns as schematized in Figure 1. A final flow of nC7 is used to prepare the columns for the next sample.

The chromatograms consist of four consecutive signals corresponding to saturates + Aro1, resins, aromatics, and asphaltenes, respectively. An important advantage of the ELS detector is that it provides quantitative responses without the need for compound-specific calibration. Since the intensity of the ELS response depends on sample concentration and injected amount, all chromatograms were normalized with respect to the total integrated area, allowing direct comparison among samples. After baseline subtraction, performed using a chromatogram obtained by injecting pure DCM, the relative percentages of the SARA fractions were calculated from the integrated areas of the corresponding peaks. As previously discussed, the first chromatographic signal does not correspond exclusively to saturates but also contains the Aro1 compounds [46]. Moreover, the saturate fraction itself includes both paraffinic and naphthenic species. Consequently, this chromatographic region contains three different families of compounds, whose relative contributions were estimated by deconvolution into three Gaussian-shaped peaks, as described in detail elsewhere [37].

It should be noted that the automated HPLC-SARA procedure used in this work is an analytical method rather than a preparative fractionation procedure. A 10 µL aliquot of a 4 mg/mL asphalt binder solution was injected, corresponding to approximately 40 µg of sample per analysis. Under these conditions, the gravimetric recovery of individual SARA fractions would not be reliable, because the mass of each collected fraction would be extremely small and strongly affected by solvent evaporation, sample handling, residual solvent, and weighing uncertainty. Therefore, absolute recovery rates for each SARA fraction were not determined. The SARA values reported here are based on chromatographic peak integration after baseline subtraction and normalization of the total integrated area. They should therefore be interpreted as operational relative distributions derived from the automated HPLC response, rather than as absolute mass recoveries of isolated fractions. As generally observed for asphalt binders and heavy petroleum systems, very polar or high-molecular-weight species may interact strongly with chromatographic stationary phases, and irreversible adsorption cannot be completely excluded. However, because all samples were analysed using the same automated procedure and under identical chromatographic conditions, the method is suitable for comparing relative compositional changes along the aging sequence. As an additional quality-control check, an asphalt binder selected as an internal reference sample is periodically analysed to verify the stability of the chromatographic response and the repeatability of the calculated SARA distribution over time. The good repeatability of replicate analyses, the periodic analysis of the internal reference sample, and the stability of the chromatographic profiles further support the reliability of the method for tracking aging-induced changes.

### 2.4. FTIR Analysis

The FTIR spectra were acquired in attenuated total reflection mode (ATR) using a PerkinElmer Spectrum 400 spectrometer equipped with a PerkinElmer Universal ATR device containing a zinc selenide (ZnSe)/diamond composite crystal (PerkinElmer, Inc., Shelton, CT, USA). The samples were analyzed in the solid state, and three measurements were acquired for each sample. Each spectrum was recorded using 16 scans, with a resolution of 4 cm^−1^ over the 650–4000 cm^−1^ spectral range. Between consecutive measurements, the ATR crystal was carefully cleaned, and a background spectrum was recorded. Spectral processing was performed using PerkinElmer Spectrum software v10.4. The same processing procedure was applied to all spectra and included ATR correction and automatic baseline correction over the complete spectral range. The automatic baseline correction algorithm identifies baseline regions by calculating a smoothed first derivative of the spectrum and then adjusts the baseline using these regions as reference points. Baseline correction and subsequent band integration were performed in absorbance units in order to preserve band intensities. No sample-specific manual selection of baseline points was applied. ATR correction was performed using the ATR correction routine available in the PerkinElmer Spectrum software and was applied to compensate for the wavelength dependence of the ATR penetration depth, which causes the effective path length to vary across the spectrum. The same ATR correction settings were used for all spectra. Since the asphalt binders are highly viscous and deformable materials, particular care was taken to ensure good contact between the sample and the ATR crystal. Before each acquisition, the preview spectrum was checked to verify that a stable and intense signal was obtained. The correction was therefore applied assuming good sample–crystal contact, with the contact correction factor set to zero. No sample-specific manual adjustment of ATR correction parameters was performed. As a sensitivity check, the spectra were also processed without ATR correction, using only the automatic baseline correction procedure, and the corresponding results are reported in the Supplementary Materials.

The carbonyl band, centered at approximately 1700 cm^−1^, was integrated over the 1660–1740 cm^−1^ range. This relatively broad interval was selected because carbonyl-containing oxidation products in aged asphalt binders may include ketones, carboxylic acids, esters, anhydrides, and other species, whose C=O stretching bands occur over a relatively wide spectral range. Therefore, *I_C=O_* should be regarded as an overall carbonyl oxidation index rather than as the contribution of a single carbonyl-containing functionality. The sulfoxide absorption is centered at approximately 1030 cm^−1^; however, following commonly adopted procedures in asphalt aging studies, the 980–1070 cm^−1^ interval was integrated to calculate the sulfoxide-related oxidation index. It should be noted that this spectral region is relatively complex and may include contributions from vibrational modes other than S=O stretching. Therefore, *I_S=O_* should be interpreted as an operational FTIR oxidation index associated with the sulfoxide region, rather than as a selective quantification of sulfoxide groups alone [14]. The integrated areas were used to calculate the carbonyl and sulfoxide indices, according to the following equations:

$$I_{C=O}=\frac{A_{1660-1740}}{A_{1350-1515}}$$

$$I_{S=O}=\frac{A_{980-1070}}{A_{1350-1515}}$$

where *A*_1660–1740_, *A*_980–1070_, and *A*_1350–1515_ represent the integrated areas of the carbonyl, sulfoxide, and reference bands, respectively. The area in the 1350–1515 cm^−1^ range, mainly corresponding to aliphatic C–H deformation modes, particularly CH_2_CH_3_ bending vibrations, was used as a reference band in accordance with commonly adopted procedures in asphalt aging studies, because they are generally considered relatively stable and less affected by aging than the oxidation bands [41,47]. Moreover, this reference region lies in the same spectral window as the carbonyl and sulfoxide bands and is therefore less affected by ATR-related differences in beam penetration depth, sample–crystal contact, and baseline correction. Nevertheless, it should be acknowledged that this reference region may also undergo minor changes during severe aging; therefore, the resulting carbonyl and sulfoxide indices should be interpreted as operational FTIR oxidation indices rather than as absolute concentrations of oxygen-containing functional groups [13,48].

**Figure 2:**
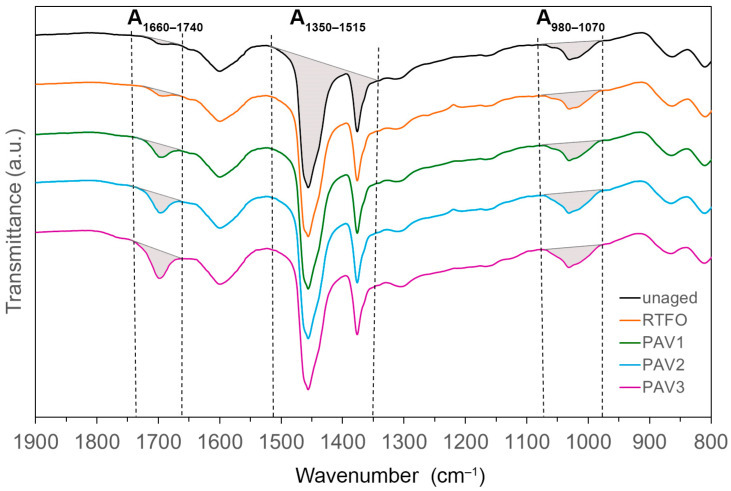
FTIR spectra of the 50–70 asphalt binders at the different aging stages.

For illustrative purposes, Figure 2 reports a selected region of the infrared spectra of the 50–70 asphalt binder at different aging stages, showing the integration intervals and the characteristic absorption bands used to determine the carbonyl and sulfoxide indices.

### 2.5. Statistical Analysis

One-way analysis of variance (ANOVA) was performed separately for aromatics, asphaltenes, and the colloidal instability index (*I_c_*) to assess whether the overall differences observed among aging conditions were larger than the variability associated with replicate HPLC measurements. When ANOVA indicated statistically significant differences, Tukey’s honestly significant difference (HSD) post-hoc test was applied to identify which aging conditions differed from each other. Attention was given to pairwise comparisons between successive aging conditions, namely unaged–RTFO, RTFO–PAV1, PAV1–PAV2, and PAV2–PAV3. Statistical significance was evaluated at *p* < 0.05.

The aging conditions considered in this work, namely unaged, RTFO, PAV1, PAV2, and PAV3, were treated as discrete categorical states rather than as equally spaced points on a quantitative aging scale. Therefore, the ANOVA and Tukey HSD post-hoc analyses were not intended to describe aging kinetics or to assume linear increments in aging severity, but only to evaluate whether the observed compositional differences among the investigated aging conditions were statistically larger than the variability associated with replicate measurements.

## 3. Results and Discussion

Since the aim of this work is to monitor compositional changes induced by aging, it is essential to verify that the observed variations are significantly larger than the experimental uncertainty associated with the measurements. Therefore, five replicate analyses were performed for each asphalt binder and aging condition. The percentage uncertainty (%U) was calculated as

$$\%U=\frac{V_{max}-V_{min}}{V_{av}}\times 100$$

where *V_max_*, *V_min_* and *V_av_*, represent the maximum, minimum, and average values obtained from the replicate measurements, respectively. For all SARA fractions and aging levels, %*U* was generally below 2–3%, substantially lower than the compositional variations observed during aging. The average values obtained for all samples are reported in Table 2 as mean ± standard deviation (σ).

The low standard deviations reported in Table 2 confirm the limited dispersion of the experimental data and, consequently, the good repeatability of the HPLC-SARA method. The compositions of the unaged asphalt binders are consistent with their penetration grade. As asphalt binder softness increases, the asphaltene content decreases, whereas no clear trend is observed for the other SARA fractions. Similarly, the colloidal instability index exhibits comparable values for the four unaged asphalt binders, suggesting that differences in penetration grade are not directly reflected in the initial colloidal stability.

**Figure 3:**
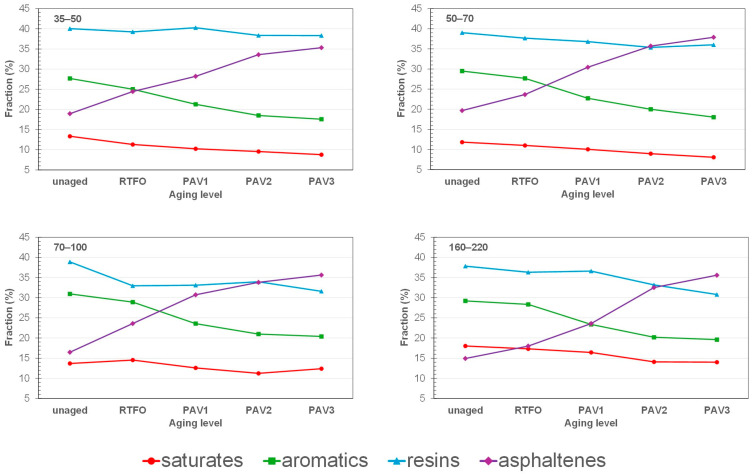
Evolution of SARA fraction with aging for the four asphalt binders. Data are reported as mean values of five replicate HPLC analyses.

The evolution of SARA fractions during artificial aging follows the well-established trends reported for thermo-oxidative aging of asphalt binders. For all asphalt binders and aging levels, aromatics progressively decrease while asphaltenes increase, reflecting the conversion of less polar species into more polar oxidation products. Figure 3 provides a clearer visualization of these compositional changes as a function of aging level. 

The observed decrease in aromatics and increase in asphaltenes are consistent with the formation of more polar and less soluble material during thermo-oxidative aging [12,49]. However, this interpretation should be broadened, since the redistribution among SARA fractions during thermo-oxidative aging cannot be attributed exclusively to the formation of oxygen-containing functional groups. Although carbonyls, carboxylic acids, anhydrides, sulfoxides, and other polar oxidation products contribute to increased polarity and altered solubility, the magnitude of the observed SARA changes cannot necessarily be explained by oxygen uptake alone. Changes in apparent molecular size, hydrogen content, aromaticity, molecular association, and solvent–asphalt interactions can also affect solubility class and colloidal behavior. Accordingly, oxidative aging may shift molecules toward resin- or asphaltene-like operational classes through multiple concurrent processes, including oxidation, hydrocarbon addition, aromatization, condensation or association processes, and changes in apparent molecular size [16,17,18,19].

The saturates fraction is the least chemically reactive and is therefore only slightly affected by artificial aging. Its content remains almost constant or shows a slight decrease after RTFO and PAV1. This variation may be partly related to the loss of low-molecular-weight components during high-temperature short-term aging; however, since mass loss was not quantified separately, no correction was applied and no specific contribution was assigned to individual SARA fractions. Moreover, saturates are not expected to be a major source of newly formed aromatic structures under the aging conditions investigated, their direct transition into the aromatic fraction is unlikely; however, they may contribute to more polar fractions if sufficiently oxidized or involved in association processes. Conversely, the more reactive aromatic and polar fractions undergo chemical and associative transformations that modify their solubility and chromatographic behavior. As a result, part of the material initially classified within the aromatic/resin domains is progressively redistributed toward more polar and less soluble fractions. Consequently, the content of aromatics decreases, while that of asphaltenes increases, whereas the resin content may increase, decrease, or remain approximately constant. The resin fraction should therefore be considered an intermediate operational class, affected simultaneously by the formation of resin-like species from aromatics and by the transformation or association of resin-like species into asphaltene-like material. These results are in qualitative agreement with trends derived from TLC-FID analysis [7,29,34], thus confirming that the HPLC method captures the same fundamental compositional pathways.

**Figure 4:**
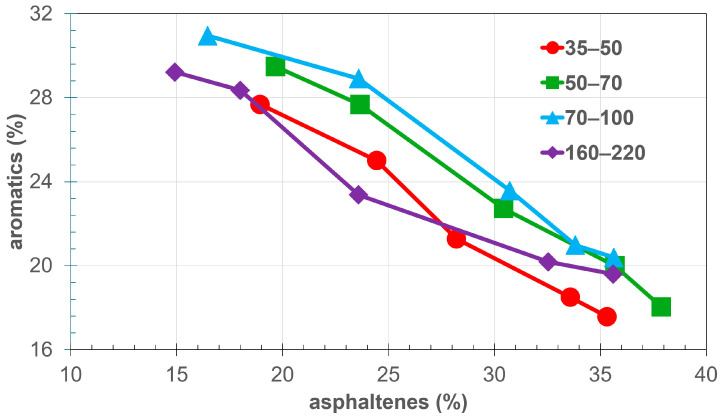
Aromatics to asphaltenes shift during aging for the four asphalt binders. Data are reported as mean values of five replicate HPLC analyses.

The relatively small variation observed at moderate aging levels (RTFO and PAV1) should not be interpreted as an absence of chemical evolution, but rather as the result of competing transformation processes involving aromatics, resins, and asphaltenes. Accordingly, for all asphalt binders, regardless of penetration grade, the resin content remains nearly constant during conventional aging (RTFO + PAV1), followed by a slight decreasing trend at higher aging levels. This behaviour suggests that, at moderate aging levels, the formation of resin-like material from less polar aromatic domains may be balanced by the simultaneous transformation, association, or condensation of resin-like species into more asphaltene-like material. At higher aging levels (PAV2 and PAV3), this apparent balance is no longer maintained, and the depletion of resins, accompanied by a significant increase in asphaltenes, indicates that the formation of less soluble asphaltene-like material becomes dominant. Figure 4 illustrates the relationship between aromatics and asphaltenes as a function of penetration grade and aging level.

**Figure 5:**
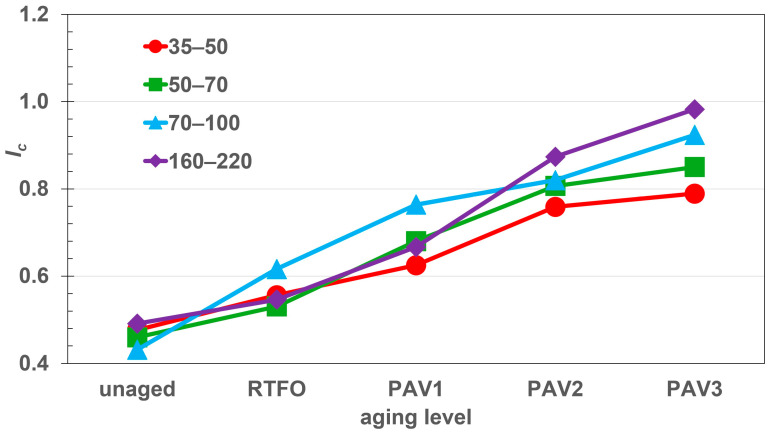
Evolution of the colloidal instability index with aging for the four asphalt binders. Data are reported as mean values of five replicate HPLC analyses.

The broadly similar evolution observed for the four asphalt binders suggests that the relative compositional pathways associated with thermo-oxidative aging are comparable across the investigated penetration grades. Although the binders differ in their initial SARA distribution, the decrease in aromatics and the associated increase in asphaltenes follow similar overall trajectories. This indicates that the aging-induced redistribution is not controlled solely by the initial abundance of each fraction. The same trend is reflected in the evolution of the colloidal instability index (*I_c_*), shown in Figure 5.

Interestingly, while the absolute contents of aromatics, resins, and asphaltenes differ among asphalt binders, the instability index follows a remarkably consistent aging trajectory. This suggests that thermo-oxidative aging follows similar colloidal pathways regardless of penetration grade. Therefore, *I_c_* may provide a more general description of aging-induced colloidal evolution than the individual fraction contents considered separately.

From a physical point of view, the progressive increase in *I_c_* indicates a reduction in the relative amount of dispersing maltene components with respect to the asphaltene-rich fraction. This compositional shift is expected to be associated with the increase in viscosity and stiffness commonly observed during asphalt binder aging, as well as with reduced relaxation ability and greater susceptibility to brittle behaviour. However, since rheological measurements were not included in the scope of the present work, *I_c_* should be interpreted as a compositional and colloidal indicator complementary to, rather than replacing, performance-related rheological parameters. Establishing quantitative relationships between HPLC-derived *I_c_* and rheological or durability-related properties represents an important objective for future work.

One-way ANOVA was performed separately for aromatics, asphaltenes, and *I_c_* using aging condition as the independent factor (Table 3). The aging conditions considered in this work, namely unaged, RTFO, PAV1, PAV2, and PAV3, were treated as discrete categorical states rather than as equally spaced points on a quantitative aging scale. Therefore, the ANOVA was not intended to describe aging kinetics or to assume linear increments in aging severity. Its purpose was limited to assessing whether the compositional differences observed among the different aging conditions were larger than the experimental variability associated with replicate HPLC measurements. Statistical significance was evaluated at *p* < 0.05; however, exact *p*-values are not reported in Table 3 because all ANOVA results were highly significant, with *p*-values far below 0.001. The ANOVA results indicate that, for each asphalt binder, the differences observed among the discrete aging conditions are larger than the variability associated with replicate measurements. These results support the repeatability of the automated HPLC-SARA workflow in distinguishing the main compositional changes induced by the applied aging treatments. However, the suitability of the method for aging assessment should not be inferred from statistical significance alone, but from the combined evidence of low replicate variability, consistent aging trends across different asphalt binders, stable chromatographic profiles, and agreement with independent FTIR-derived oxidation indicators. Since the aging conditions do not represent equally spaced time or severity increments, no kinetic interpretation was derived from this statistical analysis.

Tukey HSD post-hoc analysis was then performed to identify which aging conditions differed significantly from each other. The results showed that all successive aging steps produced statistically significant changes in asphaltenes and *I_c_* for all asphalt binders. For aromatics, all successive aging steps were significant except for the PAV2–PAV3 comparison in the 70–100 and 160–220 binders, for which the differences were not statistically significant. This indicates that the main aging-induced compositional changes are statistically supported, while the decrease in aromatics may become less pronounced at the most severe aging levels for some binders.

**Figure 6:**
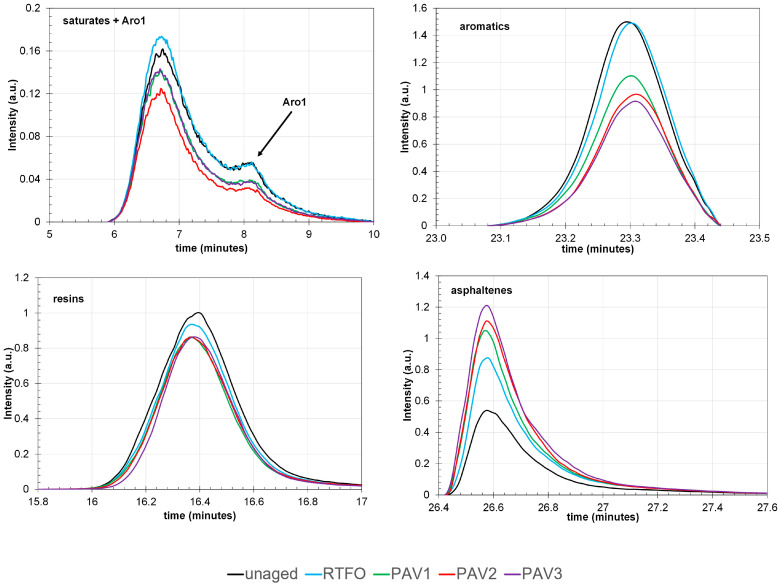
Evolution of the peaks corresponding to the four SARA fractions with aging for asphalt 70–100.

The chromatographic profiles provide additional information on the robustness of the HPLC-SARA workflow. Figure 6 shows, as a representative example, the chromatograms obtained for asphalt binder 70–100 at the different aging conditions. The comparison of peak shapes and signal profiles allows a qualitative assessment of chromatographic stability throughout the aging sequence and provides further evidence that the observed compositional variations are associated with aging rather than with changes in chromatographic performance. In these chromatograms, as well as in those corresponding to the other asphalt binders (not shown for the sake of brevity), the peaks associated with the four fractions maintain a consistent shape throughout the aging sequence, with no evidence of signal distortion, peak broadening, or chromatographic drift. This observation confirms that the compositional variations discussed above are not accompanied by changes in chromatographic performance and therefore reflect actual aging-induced transformations of the asphalt binder.

The apparently higher noise observed in the saturates region is mainly a consequence of its lower relative abundance, which results in a smaller vertical scale. The chromatographic profiles also provide a qualitative confirmation of the trends obtained from peak integration. In particular, the saturates and Aro1 signals remain relatively unchanged during short-term aging, whereas the aromatic fraction exhibits a progressive reduction as aging advances. The relatively small variation in the resin peak is also consistent with the trends obtained from peak integration.

To further assess whether the colloidal destabilization derived from HPLC-SARA analysis is chemically consistent with oxidative aging, two FTIR-derived indices were also evaluated.

The complete set of FTIR oxidation indices calculated for all asphalt binders and aging conditions is reported in Table 4. Representative ATR-FTIR spectra for each asphalt binder series are provided in the Supplementary Materials (Figures S1, S3, S5 and S7). For each binder, both the full spectral range and a zoom of the diagnostic region used for calculating the carbonyl and sulfoxide indices are reported. For clarity, here only one representative spectrum is shown for each aging condition, whereas the quantitative indices reported in Table 4 were calculated from three replicate FTIR measurements.

As an additional sensitivity check, the carbonyl and sulfoxide indices were also calculated from spectra processed without ATR correction, using only the automatic baseline correction procedure. The corresponding spectra are reported in the Supplementary Materials for each asphalt binder series (Figures S2, S4, S6 and S8), together with a zoom of the diagnostic spectral region used for band integration. A supplementary table reports the carbonyl and sulfoxide indices calculated from the average integrated areas obtained from these non-ATR-corrected spectra (Table S1), and an additional supplementary figure shows the evolution of these indices as a function of aging condition (Figure S9). Although the absolute numerical values differed from those obtained after ATR correction, the aging trends were preserved, confirming that the interpretation of the FTIR evolution was not dependent on the ATR correction step.

**Figure 7:**
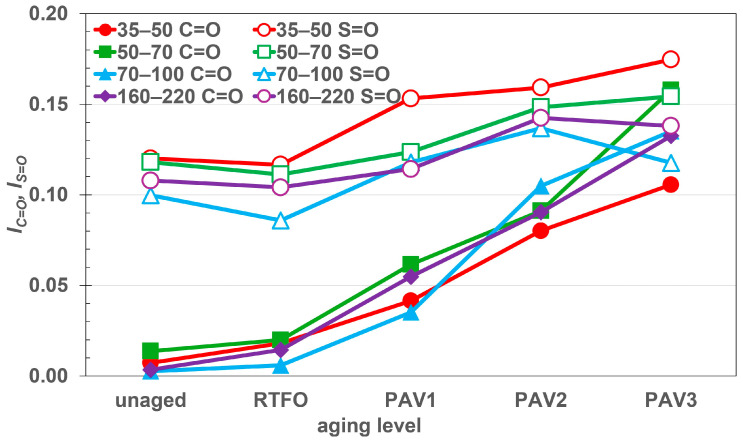
Carbonyl and sulfoxide indices obtained by FTIR for the investigated asphalt binders at different aging levels. Data are reported as mean values of three replicate FTIR measurements.

Figure 7 shows the evolution of *I_C=O_* and *I_S=O_* for the four asphalt binders as a function of aging condition.

**Figure 8:**
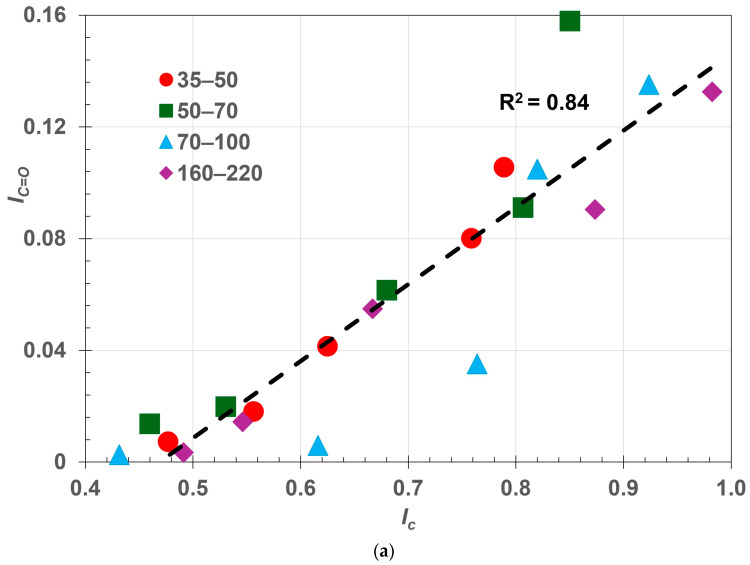

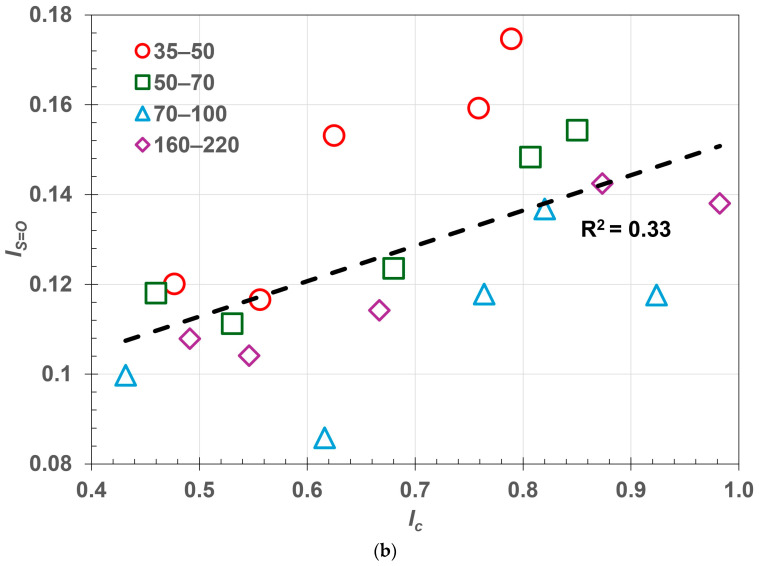
(**a**) Correlation between *I_c_* and *I_C=O_* for the investigated asphalt binders at different aging levels. The dashed line represents the linear regression calculated using all binders and aging conditions. (**b**) Correlation between *I_c_* and *I_S=O_* for the asphalt binders investigated at different aging levels. The dashed line represents the linear regression calculated using all asphalt binders and aging conditions.

Both indices progressively increase with aging, confirming the formation of oxidation products during thermo-oxidative aging. In particular, the carbonyl index exhibits a marked increase after long-term aging, in agreement with the progressive decrease in aromatics and increase in asphaltenes observed by HPLC-SARA analysis. Figure 8 compares the evolution of the FTIR-derived oxidation indices with the colloidal instability index. As shown in Figure 8a, *I_C=O_* exhibits a strong positive correlation with *I_c_*, indicating that the increase in colloidal instability is closely associated with the oxidation processes responsible for the formation of oxygen-containing polar functionalities. The sulfoxide index also shows an overall increasing trend, although with larger scatter and some non-monotonic variations, particularly at short-term aging levels (Figure 8b).

This different behaviour can also be related to the different spectral selectivity of the two integration regions. The relatively broad carbonyl interval accounts for the presence of different carbonyl-containing oxidation products, including ketones, carboxylic acids, esters, and anhydrides, whose C=O stretching bands may occur at slightly different wavenumbers. In contrast, the sulfoxide absorption is intrinsically narrower and centered at approximately 1030 cm^−1^, whereas the integrated 980–1070 cm^−1^ region is spectrally more complex and may include additional contributions not directly related to S=O stretching. Therefore, *I_C=O_* can be considered a more robust overall oxidation marker, while *I_S=O_* should be interpreted as an operational sulfoxide-related FTIR index. This lower spectral selectivity likely contributes, together with asphalt binder-dependent sulfur chemistry, to the larger scatter observed in the *I_c_*–*I_S=O_* correlation. 

This behaviour is consistent with previous studies reporting that sulfoxide formation is generally more dependent on asphalt binder source and initial sulfur content than carbonyl formation, resulting in a less uniform evolution during aging [12,14]. Consequently, carbonyl formation appears to be more systematically associated with the compositional evolution captured by HPLC-SARA analysis, whereas sulfoxide formation may be more strongly influenced by asphalt binder-specific characteristics.

Considering all asphalt binders and aging conditions, *I_c_* showed a strong correlation with the carbonyl index (*R*^2^ = 0.84), whereas the correlation with the sulfoxide index was weaker (*R*^2^ = 0.33). This correlation also remains strong when considered separately for the different penetration grades, as shown in Table 5.

Overall, the strong correlation observed between the colloidal instability index and the FTIR-derived carbonyl index supports the use of automated HPLC-SARA analysis as a reliable tool for monitoring aging-induced compositional evolution and colloidal destabilization. It should be emphasized that this correlation does not imply that carbonyl formation alone controls the SARA redistribution. Rather, *I_C=O_* should be regarded as an oxidation-sensitive marker that evolves in parallel with a broader set of aging-induced transformations, including oxygen incorporation, hydrocarbon addition, aromatization, condensation or association processes, and changes in apparent molecular size. Therefore, the strong *I_c_*–*I_C=O_* correlation indicates that colloidal destabilization and chemical oxidation progress consistently during aging, even though the underlying redistribution among SARA fractions is governed by multiple concurrent mechanisms.

## 4. Conclusions

This work investigated the evolution of SARA fractions in asphalt binders subjected to progressive thermo-oxidative aging using an automated HPLC-SARA workflow. The results obtained for four penetration-grade asphalt binders subjected to multiple aging levels revealed consistent and well-defined compositional trends. The main conclusions can be summarized as follows:

(1) The automated HPLC-SARA workflow provided repeatable compositional data for all asphalt binders and aging conditions investigated. The percentage uncertainty associated with replicate HPLC analyses was generally below 2–3%, confirming that the method is suitable for detecting aging-induced compositional variations larger than the experimental variability.
(2) In agreement with the established understanding of asphalt binder oxidation, aromatics progressively decreased while asphaltenes increased, reflecting the formation of more polar species. For example, asphaltenes increased from 18.9% to 35.4% for the 35–50 binder, from 19.8% to 37.9% for the 50–70 binder, from 16.5% to 35.6% for the 70–100 binder, and from 14.9% to 35.6% for the 160–220 binder between the unaged and PAV3 conditions. Saturates exhibited only minor variations, which may be partly associated with the possible loss of lighter components and with relative changes in the distribution of the other SARA fractions.
(3) The resin fraction behaved as an intermediate operational class. It remained relatively stable at moderate aging levels and then decreased at more advanced aging stages, consistent with a dynamic balance between the formation of resin-like material and its transformation, association, or condensation into more asphaltene-like material.
(4) The qualitative agreement with the extensive body of literature based on TLC-FID and other chromatographic methods confirms that automated HPLC-SARA analysis can capture the key compositional pathways associated with asphalt aging. At the same time, the automated workflow provides important practical advantages, including reduced operator dependence, short analysis times, controlled solvent consumption, and high repeatability, making it particularly suitable for systematic investigations involving multiple asphalt binders and aging conditions.
(5) The results further indicate that thermo-oxidative aging follows broadly similar compositional pathways across asphalt binders with different penetration grades. The systematic increase in *I_c_* confirms that this parameter can be used as a composition-based descriptor of aging-induced colloidal destabilization.
(6) The HPLC-derived colloidal instability index showed a strong correlation with the FTIR carbonyl index, with R^2^ = 0.84 when all asphalt binders and aging conditions were considered. In contrast, the correlation with the sulfoxide index was weaker overall, with R^2^ = 0.33. This indicates that the colloidal evolution captured by HPLC-SARA is chemically consistent with independent FTIR oxidation markers, especially carbonyl formation.
(7) Overall, the findings demonstrate that automated HPLC-SARA represents a valuable complementary tool for aging assessment. Compared with conventional SARA fractionation methods, the automated workflow offers reduced operator dependence, controlled solvent consumption, and short analysis times, making it particularly suitable for comparative studies involving multiple asphalt binders and aging conditions. The integration of HPLC-SARA analysis with FTIR oxidation indicators further enhances its applicability by linking compositional evolution to the underlying chemical processes responsible for aging. Future work should extend the approach to modified, recycled, and rejuvenated asphalt binders and further investigate the relationships between compositional evolution, colloidal stability, and performance-related properties.

## Figures and Tables

**Table 1:** Main physical properties of the unaged asphalt binders and aging conditions investigated in this study.

Penetration Grade	Penetration at 25 °C [dmm]	Softening Point [°C]	PG
35–50	40	52.3	70–22
50–70	64	49.3	58–16
70–100	95	44.5	58–16
160–220	190	35.9	46–28

**Table 2:** SARA fractions from HPLC for unaged and aged asphalt samples (average value ± σ) and colloidal instability index.

Penetration Grade	Aging Condition	Saturates	Aromatics	Resins	Asphaltenes	*I_c_*
35–50	unaged	13.3 ± 0.17	27.7 ± 0.30	40.1 ± 0.58	18.9 ± 0.11	0.477 ± 5.49 × 10^−3^
	RTFO	11.3 ± 0.13	25.0 ± 0.39	39.3 ± 0.33	24.4 ± 0.28	0.556 ± 3.55 × 10^−3^
	PAV1	10.2 ± 0.12	21.3 ± 0.13	40.3 ± 0.40	28.2 ± 0.37	0.625 ± 5.17 × 10^−3^
	PAV2	9.6 ± 0.09	18.5 ± 0.14	38.3 ± 0.45	33.6 ± 0.32	0.759 ± 7.77 × 10^−3^
	PAV3	8.7 ± 0.11	17.6 ± 0.24	38.3 ± 0.54	35.4 ± 0.37	0.789 ± 9.83 × 10^−3^
50–70	unaged	11.8 ± 0.15	29.5 ± 0.28	38.9 ± 0.42	19.8 ± 0.16	0.461 ± 2.54 × 10^−3^
	RTFO	11.0 ± 0.11	27.7 ± 0.39	37.7 ± 0.55	23.6 ± 0.28	0.529 ± 1.00 × 10^−2^
	PAV1	10.0 ± 0.14	22.7 ± 0.24	36.8 ± 0.51	30.5 ± 0.46	0.680 ± 6.98 × 10^−3^
	PAV2	9.0 ± 0.11	20.1 ± 0.13	35.3 ± 0.44	35.6 ± 0.39	0.807 ± 1.23 × 10^−2^
	PAV3	8.1 ± 0.12	18.0 ± 0.17	36.0 ± 0.41	37.9 ± 0.23	0.855 ± 3.99 × 10^−3^
70–100	unaged	13.7 ± 0.17	31.0 ± 0.22	38.8 ± 0.39	16.5 ± 0.23	0.431 ± 5.01 × 10^−3^
	RTFO	14.5 ± 0.14	28.9 ± 0.43	33.0 ± 0.56	23.6 ± 0.35	0.617 ± 1.04 × 10^−2^
	PAV1	12.5 ± 0.18	23.7 ± 0.17	33.1 ± 0.44	30.7 ± 0.47	0.763 ± 1.46 × 10^−2^
	PAV2	11.2 ± 0.12	21.0 ± 0.22	34.0 ± 0.37	33.8 ± 0.45	0.820 ± 5.89 × 10^−3^
	PAV3	12.4 ± 0.14	20.4 ± 0.26	31.6 ± 0.30	35.6 ± 0.46	0.926 ± 1.56 × 10^−2^
160–220	unaged	18.1 ± 0.28	29.2 ± 0.49	37.8 ± 0.33	14.9 ± 0.22	0.490 ± 7.31 × 10^−3^
	RTFO	17.3 ± 0.23	28.4 ± 0.42	36.3 ± 0.41	18.0 ± 0.21	0.546 ± 5.97 × 10^−3^
	PAV1	16.4 ± 0.16	23.4 ± 0.34	36.6 ± 0.49	23.6 ± 0.31	0.667 ± 4.74 × 10^−3^
	PAV2	14.1 ± 0.14	20.2 ± 0.28	33.2 ± 0.28	32.5 ± 0.46	0.877 ± 1.27 × 10^−2^
	PAV3	14.0 ± 0.12	19.6 ± 0.31	30.8 ± 0.38	35.6 ± 0.54	0.980 ± 7.95 × 10^−3^

**Table 3:** One-way ANOVA performed on aromatics, asphaltenes, and colloidal instability index (*I_c_*) to compare the discrete aging conditions investigated in this study. Aging conditions were treated as a categorical factor.

Asphalt Binder	Variable	*F*-Value
35–50	Aromatics	1390
35–50	Asphaltenes	2340
35–50	*I_c_*	2285
50–70	Aromatics	1786
50–70	Asphaltenes	2897
50–70	*I_c_*	1826
70–100	Aromatics	1496
70–100	Asphaltenes	1945
70–100	*I_c_*	1832
160–220	Aromatics	708
160–220	Asphaltenes	2905
160–220	*I_c_*	3057

**Table 4:** Carbonyl and sulfoxide oxidation indices calculated from ATR-FTIR spectra for all asphalt binders and aging conditions. The carbonyl index was obtained by integrating the 1660–1740 cm^−1^ region, whereas the sulfoxide index was obtained by integrating the 980–1070 cm^−1^ region, both normalized to the 1350–1515 cm^−1^ reference region. Values were calculated from the average integrated areas obtained from three replicate FTIR spectra recorded for each sample.

Asphalt Binder	Aging Condition	*I_C=O_*	*I_S=O_*
35–50	unaged	0.01	0.12
35–50	RTFO	0.02	0.12
35–50	PAV1	0.04	0.15
35–50	PAV2	0.08	0.16
35–50	PAV3	0.11	0.17
50–70	unaged	0.01	0.12
50–70	RTFO	0.02	0.11
50–70	PAV1	0.06	0.12
50–70	PAV2	0.09	0.15
50–70	PAV3	0.16	0.15
70–100	unaged	0.00	0.10
70–100	RTFO	0.01	0.09
70–100	PAV1	0.04	0.12
70–100	PAV2	0.10	0.14
70–100	PAV3	0.14	0.12
160–220	unaged	0.00	0.11
160–220	RTFO	0.01	0.10
160–220	PAV1	0.05	0.11
160–220	PAV2	0.09	0.14
160–220	PAV3	0.13	0.14

**Table 5:** Coefficients of determination obtained from the linear regressions between the colloidal instability index (*I_c_*) and the FTIR-derived carbonyl and sulfoxide indices for each asphalt binder.

Asphalt Binder	R^2^ (*I_c_* vs. *I_C=O_*)	R^2^ (*I_c_* vs. *I_S=O_*)
35–50	0.96	0.85
50–70	0.88	0.86
70–100	0.78	0.46
160–220	0.98	0.89

## Data Availability

The original contributions presented in this study are included in the article/Supplementary Material. Further inquiries can be directed to the corresponding authors.

## Supplementary Materials

The following supporting information can be downloaded at: https://www.mdpi.com/article/10.3390/ma19153249/s1, Figure S1. Representative ATR-FTIR spectra of the 35–50 asphalt binder at different aging stages, processed with ATR correction and automatic baseline correction: (a) full spectral range; (b) zoom of the diagnostic region used for the calculation of the carbonyl and sulfoxide indices. For clarity, one representative spectrum is shown for each aging condition; Figure S2. Representative ATR-FTIR spectra of the 35–50 asphalt binder at different aging stages without ATR correction: (a) full spectral range; (b) zoom of the diagnostic region used for the calculation of the carbonyl and sulfoxide indices. For clarity, one representative spectrum is shown for each aging condition. All spectra were processed only by automatic baseline correction; Figure S3. Representative ATR-FTIR spectra of the 50–70 asphalt binder at different aging stages, processed with ATR correction and automatic baseline correction: (a) full spectral range; (b) zoom of the diagnostic region used for the calculation of the carbonyl and sulfoxide indices. For clarity, one representative spectrum is shown for each aging condition; Figure S4. Representative ATR-FTIR spectra of the 50-70 asphalt binder at different aging stages, without ATR correction: (a) full spectral range; (b) zoom of the diagnostic region used for the calculation of the carbonyl and sulfoxide indices. For clarity, one representative spectrum is shown for each aging condition. All spectra were processed only by automatic baseline correction; Figure S5. Representative ATR-FTIR spectra of the 70–100 asphalt binder at different aging stages, processed with ATR correction and automatic baseline correction: (a) full spectral range; (b) zoom of the diagnostic region used for the calculation of the carbonyl and sulfoxide indices. For clarity, one representative spectrum is shown for each aging condition; Figure S6. Representative ATR-FTIR spectra of the 70–100 asphalt binder at different aging stages, without ATR correction: (a) full spectral range; (b) zoom of the diagnostic region used for the calculation of the carbonyl and sulfoxide indices. For clarity, one representative spectrum is shown for each aging condition. All spectra were processed only by automatic baseline correction; Figure S7. Representative ATR-FTIR spectra of the 160–220 asphalt binder at different aging stages, processed with ATR correction and automatic baseline correction: (a) full spectral range; (b) zoom of the diagnostic region used for the calculation of the carbonyl and sulfoxide indices. For clarity, one representative spectrum is shown for each aging condition; Figure S8. Representative ATR-FTIR spectra of the 160–220 asphalt binder at different aging stages, without ATR correction: (a) full spectral range; (b) zoom of the diagnostic region used for the calculation of the carbonyl and sulfoxide indices. For clarity, one representative spectrum is shown for each aging condition. All spectra were processed only by automatic baseline correction; Figure S9. Carbonyl and sulfoxide indices calculated from ATR-FTIR spectra processed without ATR correction and using only automatic baseline correction for the investigated asphalt binders at different aging stages. Values were calculated from the average integrated areas obtained from three replicate FTIR spectra recorded for each sample; Table S1. Carbonyl and sulfoxide oxidation indices calculated from ATR-FTIR spectra processed without ATR correction and using only automatic baseline correction. The carbonyl index was obtained by integrating the 1660–1740 cm^−1^ region, whereas the sulfoxide index was obtained by integrating the 980–1070 cm^−1^ region, both normalized to the 1350–1515 cm^−1^ reference region. Values were calculated from the average integrated areas obtained from three replicate FTIR spectra recorded for each sample.

## References

[1] Siroma R.S., Nguyen M.L., Hornych P., Chailleux E. (2020). A Literature Review of Bitumen Aging: From Laboratory Procedures to Field Evaluation. Proceedings of the ISBM2020—RILEM International Symposium on Bituminous Materials, Lyon, France, 14–16 December 2020.

[2] Airey G.D. (2003). State of the Art Report on Ageing Test Methods for Bituminous Pavement Materials. Int. J. Pavement Eng..

[3] Madeira N.C.L., Lacerda V., Romão W. (2022). Characterization of Asphalt Aging by Analytical Techniques: A Review on Progress and Perspectives. Energy Fuels.

[4] Sakanlou F., Riccardi C., Losa M. (2025). Comprehensive Review of Aging Phenomena in Conventional and Bio-Based Asphalt Binders: Challenges and Future Directions. J. Road Eng..

[5] Ahmad M., Khedmati M., Mensching D., Hofko B., Haghshenas H.F. (2024). Aging Characterization of Asphalt Binders through Multi-Aspect Analyses: A Critical Review. Fuel.

[6] Hu Y., Si W., Kang X., Xue Y., Wang H., Parry T., Airey G.D. (2022). State of the Art: Multiscale Evaluation of Bitumen Ageing Behaviour. Fuel.

[7] Zhen T., Kang X., Liu J., Zhang B., Si W., Ling T. (2023). Multiscale Evaluation of Asphalt Aging Behaviour: A Review. Sustainability.

[8] Wang F., Xiao Y., Peide C., Juntao L., Mingliang L., Zongwu C. (2020). Correlation of Asphalt Performance Indicators and Aging Degrees: A Review. Constr. Build. Mater..

[9] Sirin O., Paul D.K., Kassem E. (2018). State of the Art Study on Aging of Asphalt Mixtures and Use of Antioxidant Additives. Adv. Civ. Eng..

[10] Bhatt B., Wu S. (2025). A Comprehensive State-of-Art Review on the Use of Rejuvenators in Asphalt Pavement. J. Road Eng..

[11] Mirwald J., Werkovits S., Camargo I., Maschauer D., Hofko B., Grothe H. (2020). Investigating Bitumen Long-Term-Ageing in the Laboratory by Spectroscopic Analysis of the SARA Fractions. Constr. Build. Mater..

[12] Petersen J.C. (2009). A Review of the Fundamentals of Asphalt Oxidation: Chemical, Physicochemical, Physical Property, and Durability Relationships. Transportation Research Circular E-C140.

[13] Marsac P., Piérard N., Porot L., Van Den Bergh W., Grenfell J., Mouillet V., Pouget S., Besamusca J., Farcas F., Gabet T. (2014). Potential and Limits of FTIR Methods for Reclaimed Asphalt Characterisation. Mater. Struct..

[14] Primerano K., Mirwald J., Lohninger J., Hofko B. (2023). Characterization of Long-Term Aged Bitumen with FTIR Spectroscopy and Multivariate Analysis Methods. Constr. Build. Mater..

[15] Corbett L.W. (1969). Composition of Asphalt Based on Generic Fractionation, Using Solvent Deasphaltening, Elution-Adsorption Chromatography, and Densimetric Characterization. Anal. Chem..

[16] Siddiquee M.N., De Klerk A. (2014). Hydrocarbon Addition Reactions during Low-Temperature Autoxidation of Oilsands Bitumen. Energy Fuels.

[17] Werkovits S., Bacher M., Mirwald J., Theiner J., Rosenau T., Hofko B., Grothe H. (2023). The Impact of Field Ageing on Molecular Structure and Chemistry of Bitumen. Fuel.

[18] Wiehe I.A. (1992). A Solvent-Resid Phase Diagram for Tracking Resid Conversion. Ind. Eng. Chem. Res..

[19] Malinowski S. (2023). Aromatisation Process as Part of Bitumen Ageing in the Light of Electronic Structure and Further Oxidation of Its Components. Constr. Build. Mater..

[20] Wiehe I.A. (2008). Process Chemistry of Petroleum Macromolecules.

[21] (2018). Standard Test Method for Separation of Asphalt into Four Fractions.

[22] (2019). Standard Test Method for Characteristic Groups in Rubber Extender and Processing Oils and Other Petroleum-Derived Oils by the Clay-Gel Absorption Chromatographic Method.

[23] Karlsen D.A., Larter S.R. (1991). Analysis of Petroleum Fractions by TLC-FID: Applications to Petroleum Reservoir Description. Org. Geochem..

[24] Merusi F., Polacco G., Nicoletti A., Giuliani F. (2010). Kerosene Resistance of Asphalt Binders Modified with Crumb Rubber: Solubility and Rheological Aspects. Mater. Struct..

[25] Nciri N., Kim N., Cho N. (2021). From Street to Road: An Innovative Approach to Explore Discarded Chewing Gum as a Performance-Enhancing Modifier for Road Pavement Applications. Polymers.

[26] Nciri N., Kim N. (2022). Upcycling Discarded Shoe Polish into High Value-Added Asphalt Fluxing Agent for Use in Hot Mix Paving Applications. Materials.

[27] Saberi F., Hosseini-Barzi M. (2024). Effect of Thermal Maturation and Organic Matter Content on Oil Shale Fracturing. Int. J. Coal Sci. Technol..

[28] Wesenlund F., Grundvåg S.-A., Engelschiøn V.S., Thießen O., Pedersen J.H. (2021). Linking Facies Variations, Organic Carbon Richness and Bulk Bitumen Content—A Case Study of the Organic-Rich Middle Triassic Shales from Eastern Svalbard. Mar. Pet. Geol..

[29] Adly E., Chen S.H., Paramitha P.A., Widodo W. (2024). Laboratory Study of Aging Characteristics of Binder Warm Mix Asphalt Chemical Additive: Rutting, Fatigue Resistance, and Chemical Composition. J. Adv. Res. Appl. Mech..

[30] Budziński B., Ratajczak M., Majer S., Wilmański A. (2023). Influence of Bitumen Grade and Air Voids on Low-Temperature Cracking of Asphalt. Case Stud. Constr. Mater..

[31] Cheng X., Han S., Liu Y., Xu O. (2019). Laboratory Investigation on Low-Temperature Performance of Asphalt at Different Aging Stages. Constr. Build. Mater..

[32] Gaudenzi E., Cardone F., Lu X., Canestrari F. (2023). Chemical and Rheological Analysis of Unaged and Aged Bio-Extended Binders Containing Lignin. J. Traffic Transp. Eng. (Engl. Ed.).

[33] Gowda S., Behl A., Shanmuganathan K., Gupta A. (2025). Enhancing Asphalt Pavement Longevity Through Microcapsule-Encapsulated Rejuvenators: Mechanical Performance and Self-Healing Evaluation. Int. J. Pavement Res. Technol..

[34] Ma X., Ma X., Wang Z., Song S., Sheng Y. (2023). Investigation of Changing SARA and Fatigue Properties of Asphalt Bitumen under Ageing and Analysis of Their Relation Based upon the BP Neural Network. Constr. Build. Mater..

[35] Bissada K.K.A., Tan J., Szymczyk E., Darnell M., Mei M. (2016). Group-Type Characterization of Crude Oil and Bitumen. Part I: Enhanced Separation and Quantification of Saturates, Aromatics, Resins and Asphaltenes (SARA). Org. Geochem..

[36] Atwah I., Alsaif M., Usman M., Abualreesh M. (2025). Accelerating Saturate, Aromatic, Resin, Asphaltene (SARA) Analysis for High-Fidelity Petroleum Profiling via μSARA-HPLC. ACS Omega.

[37] Polacco G., Filippi S., Riccardi C., Leandri P., Losa M. (2026). An Automated HPLC Method for SARA Fractionation of Asphalts: Operational Assessment and Comparison with ASTM D4124. ACS Omega.

[38] Bruneau L., Tisse S., Michon L., Cardinael P. (2023). Evaluation of Asphalt Aging Using Multivariate Analysis Applied to Saturates, Aromatics, Resins, and Asphaltene Determinator Data. ACS Omega.

[39] Weigel S., Stephan D. (2018). Bitumen Characterization with Fourier Transform Infrared Spectroscopy and Multivariate Evaluation: Prediction of Various Physical and Chemical Parameters. Energy Fuels.

[40] Li J., Xing X., Hou X., Wang T., Wang J., Xiao F. (2022). Determination of SARA Fractions in Asphalts by Mid-Infrared Spectroscopy and Multivariate Calibration. Measurement.

[41] Taheri A., Khodaii A., Hajikarimi P. (2026). Correlations among Chemical and Rheological Aging Indices/Indicators of Asphalt Binder at High Temperatures. Sci. Rep..

[42] (2022). Standard Test Method for Effect of Heat and Air on a Moving Film of Asphalt Binder (Rolling Thin-Film Oven Test).

[43] (2022). Standard Practice for Accelerated Aging of Asphalt Binder Using a Pressurized Aging Vessel (PAV).

[44] Adams J.J., Schabron J.F., Boysen R. (2015). Quantitative Vacuum Distillation of Crude Oils to Give Residues Amenable to the Asphaltene Determinator Coupled with Saturates, Aromatics, and Resins Separation Characterization. Energy Fuels.

[45] Srinivasan P., Arguello E.M.E., Atwah I. (2024). Evaluating the Reliability of Solid Phase Extraction Techniques for Hydrocarbon Analysis by GC–MS. J. Chromatogr. A.

[46] Adams J.J., Rovani J.F., Planche J.-P., Loveridge J., Literati A., Shishkova I., Palichev G., Kolev I., Atanassov K., Nenov S. (2023). SAR-AD Method to Characterize Eight SARA Fractions in Various Vacuum Residues and Follow Their Transformations Occurring during Hydrocracking and Pyrolysis. Processes.

[47] Iwański M., Chomicz-Kowalska A., Maciejewski K., Iwański M.M., Radziszewski P., Liphardt A., Król J.B., Sarnowski M., Kowalski K.J., Pokorski P. (2023). Effects of Laboratory Ageing on the FTIR Measurements of Water-Foamed Bio-Fluxed Asphalt Binders. Materials.

[48] Hofko B., Porot L., Falchetto Cannone A., Poulikakos L., Huber L., Lu X., Mollenhauer K., Grothe H. (2018). FTIR Spectral Analysis of Bituminous Binders: Reproducibility and Impact of Ageing Temperature. Mater. Struct..

[49] Masson J.-F., Price T., Collins P. (2001). Dynamics of Bitumen Fractions by Thin-Layer Chromatography/Flame Ionization Detection. Energy Fuels.

