# Supplementary material for: Monitoring the Evolution of SARA Fractions During Asphalt Binder Aging by Automated HPLC and Correlation with FTIR Oxidation Indices
Source: Materials (Basel). 2026 Aug 1;19(15):3249. doi: 10.3390/ma19153249 (PMC13466625; doi:10.3390/ma19153249)
Supplement: Supplementary file 1 [file materials-19-03249-s001.zip › materials-4424499-supplementary.pdf]

The supplementary figures report representative ATR-FTIR spectra for all investigated asphalt binders and aging conditions. Figures S1, S3, S5 and S7 show spectra processed using ATR correction and automatic baseline correction, corresponding to the processing procedure used for the FTIR indices reported in Table 4 and Figure 7 of the main manuscript. Figures S2, S4, S6 and S8 report the corresponding spectra processed without ATR correction, using only the automatic baseline correction procedure. Table S1 and Figure S9 summarize the carbonyl and sulfoxide indices calculated from these non-ATR-corrected spectra.

Table S1. Carbonyl and sulfoxide oxidation indices calculated from ATR-FTIR spectra processed without ATR correction and using only automatic baseline correction. The carbonyl index was obtained by integrating the 1660–1740 cm^{-1} region, whereas the sulfoxide index was obtained by integrating the 980–1070 cm^{-1} region, both normalized to the 1350–1515 cm^{-1} reference region. Values were calculated from the average integrated areas obtained from three replicate FTIR spectra recorded for each sample.

**Asphalt Binder**	**Aging condition**	$I_{C=O}$	$I_{S=O}$
35–50	unaged	0.01	0.19
35-50	RTFO	0.01	0.19
35-50	PAV1	0.04	0.24
35-50	PAV2	0.07	0.25
35-50	PAV3	0.09	0.27
50-70	unaged	0.01	0.18
50-70	RTFO	0.02	0.18
50-70	PAV1	0.06	0.20
50-70	PAV2	0.08	0.24
50-70	PAV3	0.14	0.24
70-100	unaged	0.00	0.16
70-100	RTFO	0.01	0.14
70-100	PAV1	0.03	0.19
70-100	PAV2	0.09	0.22
70-100	PAV3	0.12	0.19
160-220	unaged	0.00	0.17
160-220	RTFO	0.01	0.17
160-220	PAV1	0.05	0.18
160-220	PAV2	0.08	0.22
160-220	PAV3	0.12	0.22

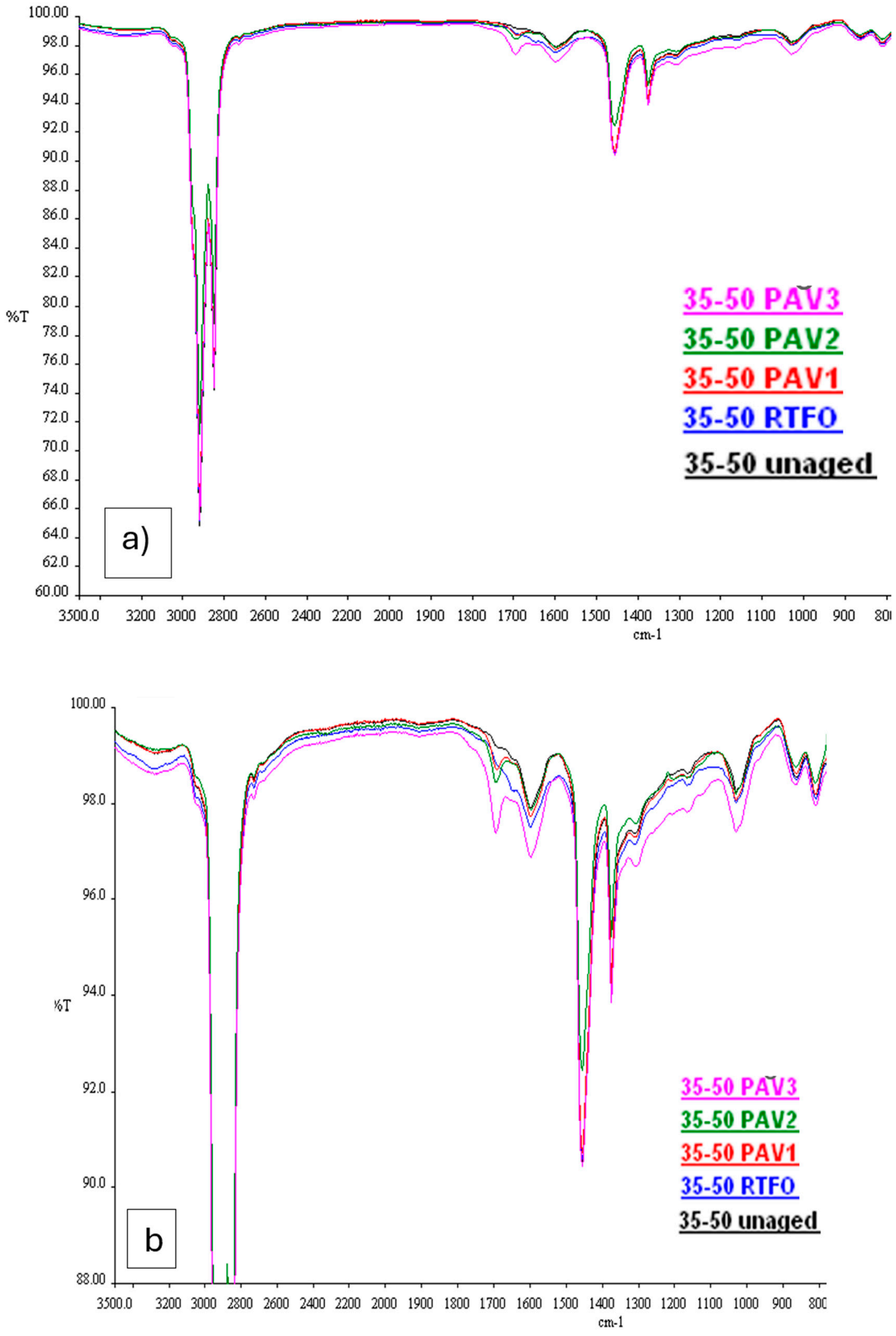

Figure S1. Representative ATR-FTIR spectra of the 35–50 asphalt binder at different aging stages, processed with ATR correction and automatic baseline correction: (a) full spectral range; (b) zoom of the diagnostic region used for the calculation of the carbonyl and sulfoxide indices. For clarity, one representative spectrum is shown for each aging condition.

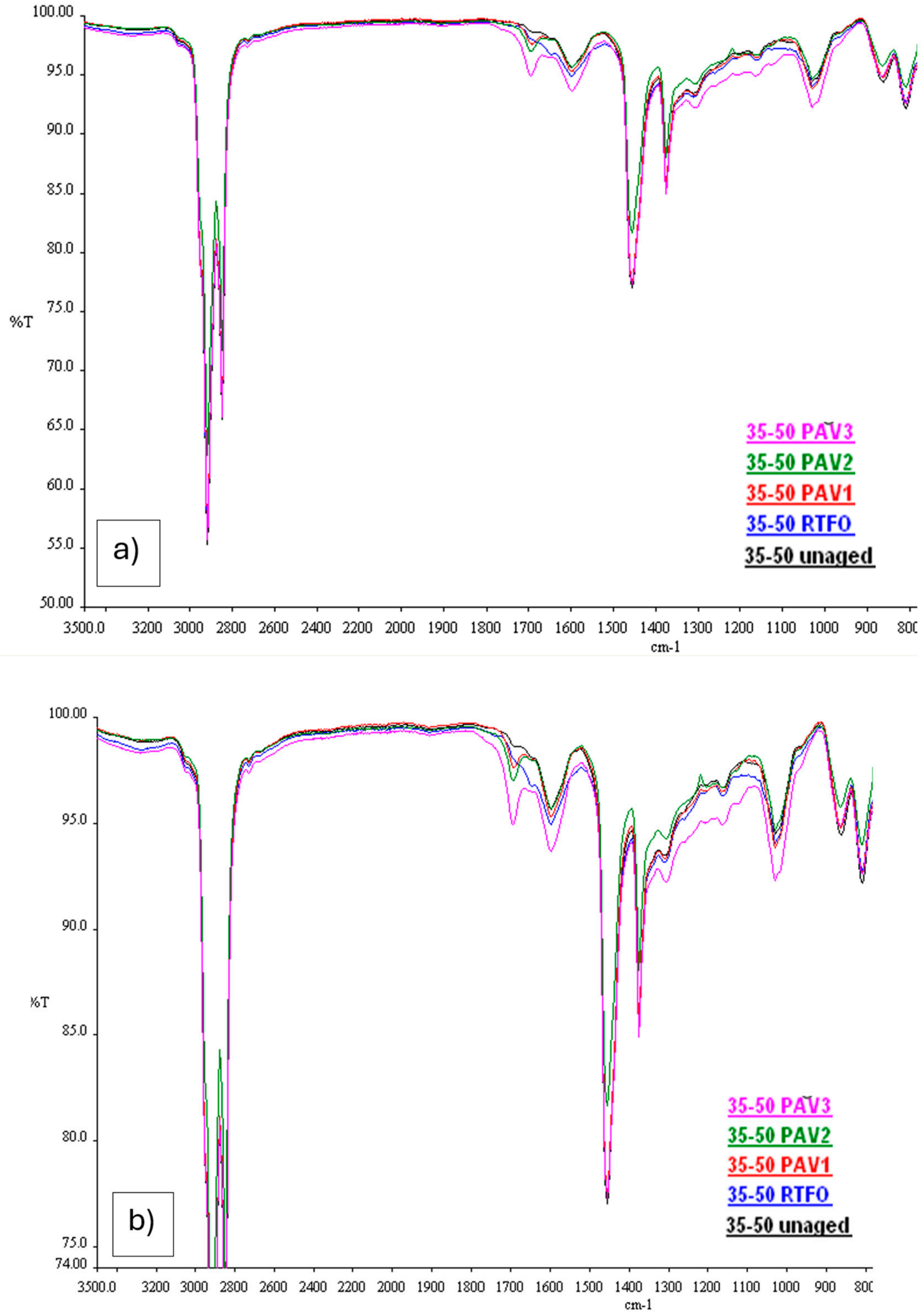

Figure S2. Representative ATR-FTIR spectra of the 35–50 asphalt binder at different aging stages without ATR correction: (a) full spectral range; (b) zoom of the diagnostic region used for the calculation of the carbonyl and sulfoxide indices. For clarity, one representative spectrum is shown for each aging condition. All spectra were processed only by automatic baseline correction.

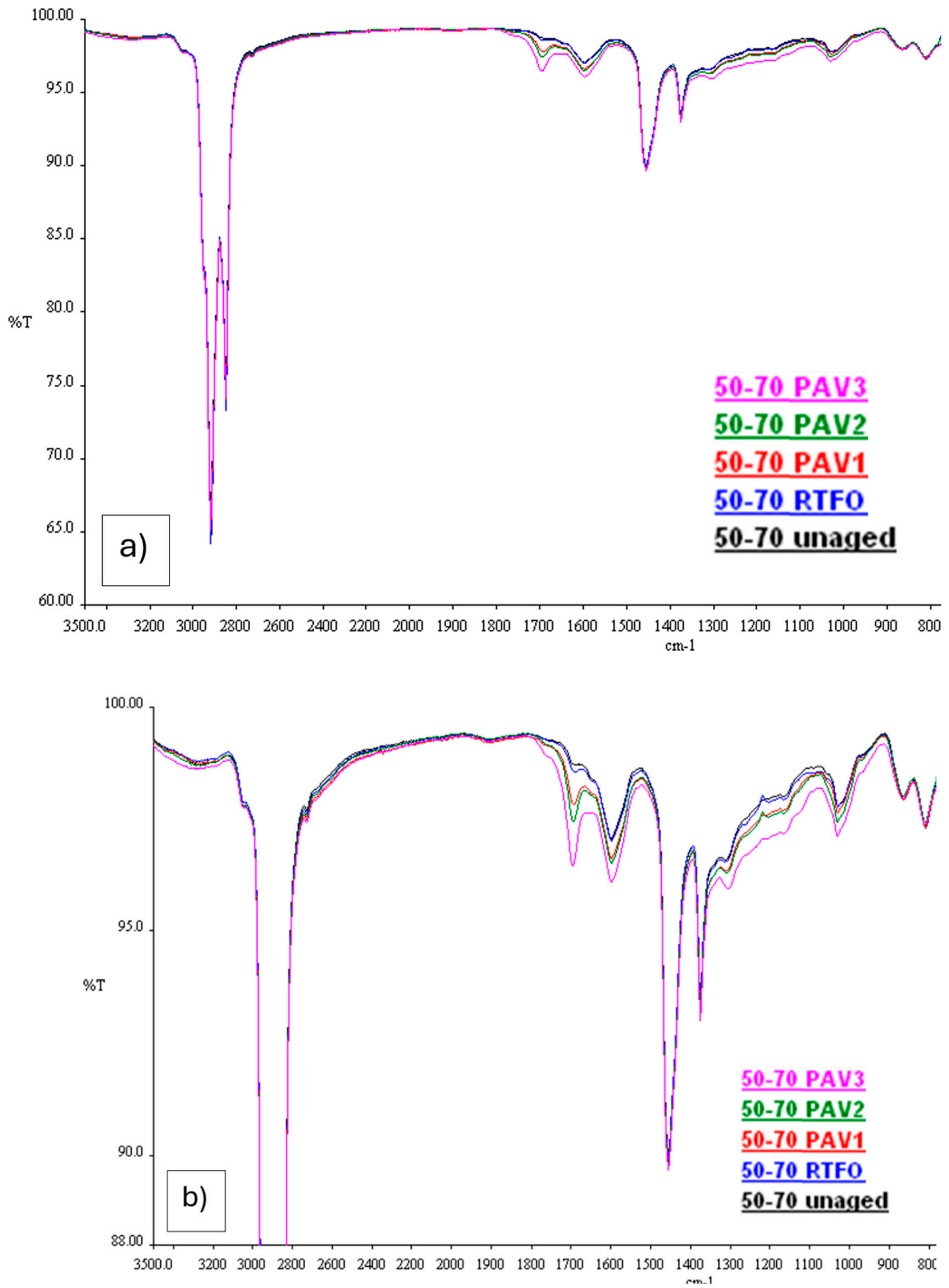

Figure S3. Representative ATR-FTIR spectra of the 50–70 asphalt binder at different aging stages, processed with ATR correction and automatic baseline correction: (a) full spectral range; (b) zoom of the diagnostic region used for the calculation of the carbonyl and sulfoxide indices. For clarity, one representative spectrum is shown for each aging condition.

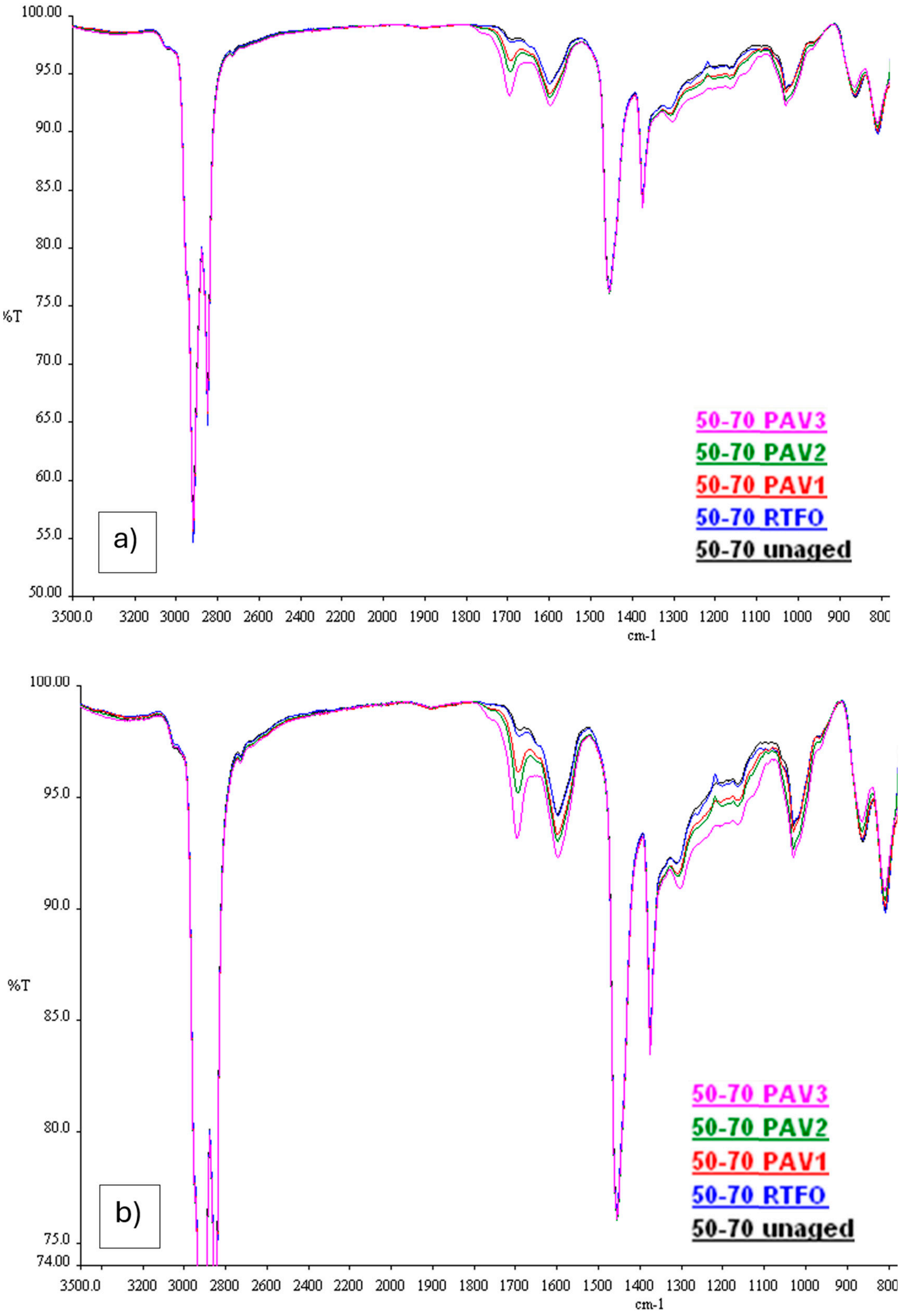

Figure S4. Representative ATR-FTIR spectra of the 50-70 asphalt binder at different aging stages, without ATR correction: (a) full spectral range; (b) zoom of the diagnostic region used for the calculation of the carbonyl and sulfoxide indices. For clarity, one representative spectrum is shown for each aging condition. All spectra were processed only by automatic baseline correction.

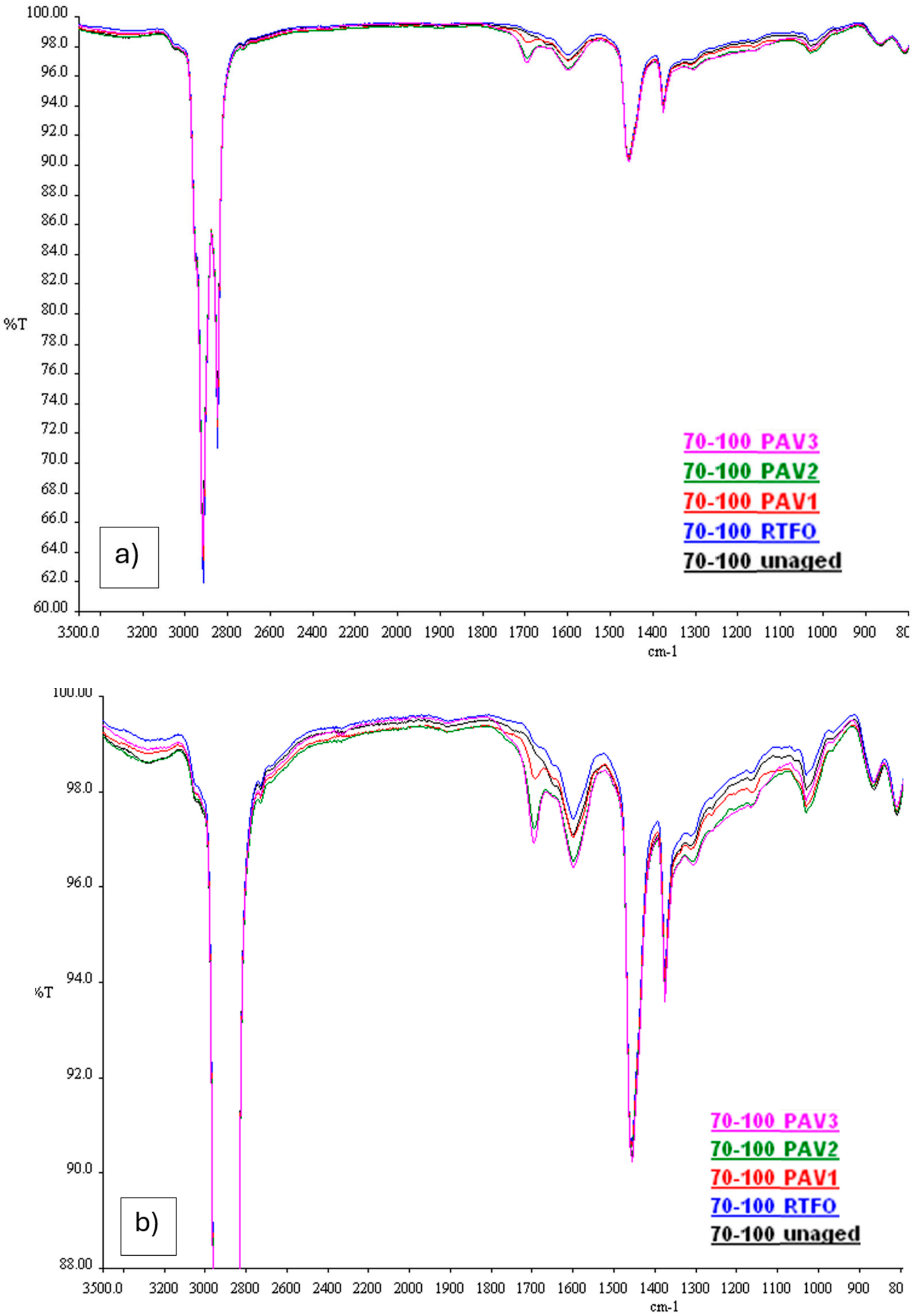

Figure S5. Representative ATR-FTIR spectra of the 70–100 asphalt binder at different aging stages, processed with ATR correction and automatic baseline correction: (a) full spectral range; (b) zoom of the diagnostic region used for the calculation of the carbonyl and sulfoxide indices. For clarity, one representative spectrum is shown for each aging condition.

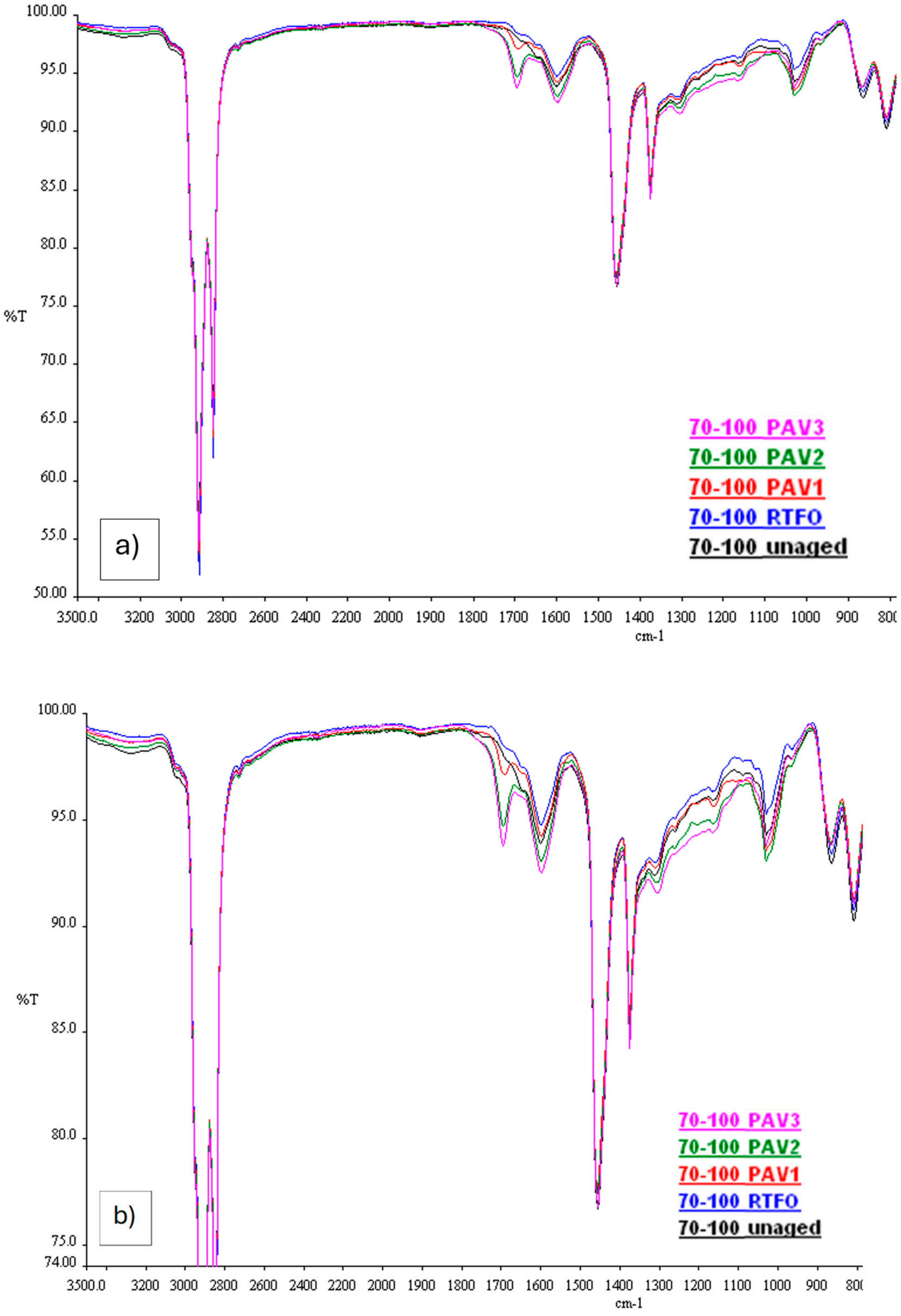

Figure S6. Representative ATR-FTIR spectra of the 70-100 asphalt binder at different aging stages, without ATR correction: (a) full spectral range; (b) zoom of the diagnostic region used for the calculation of the carbonyl and sulfoxide indices. For clarity, one representative spectrum is shown for each aging condition. All spectra were processed only by automatic baseline correction.

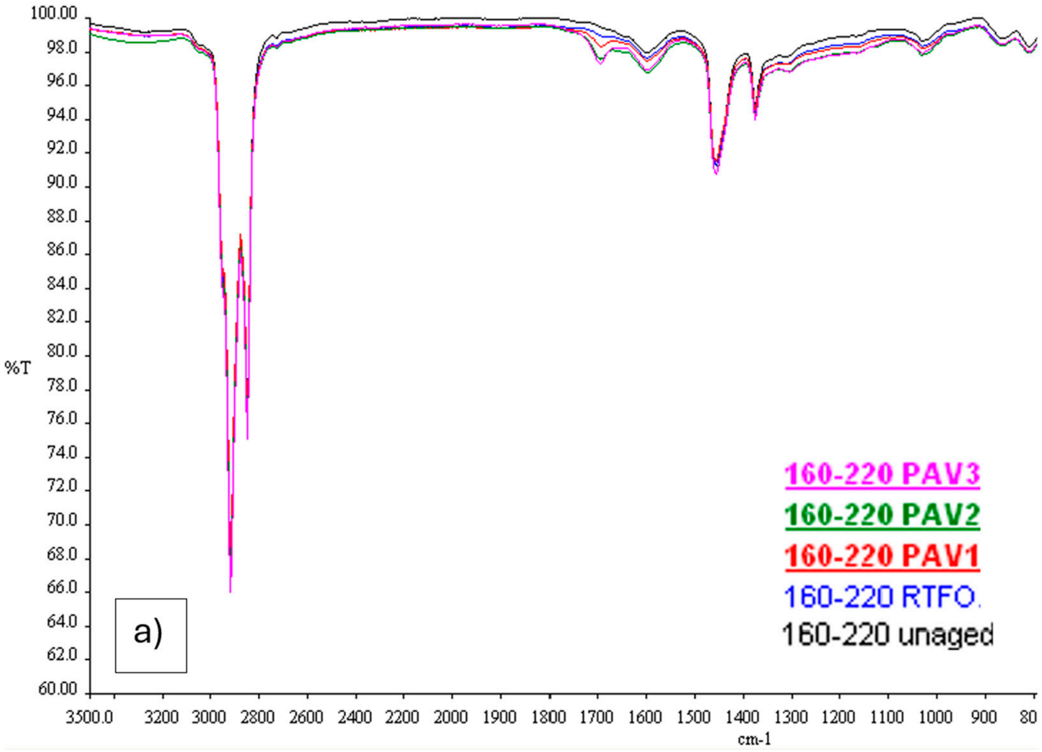

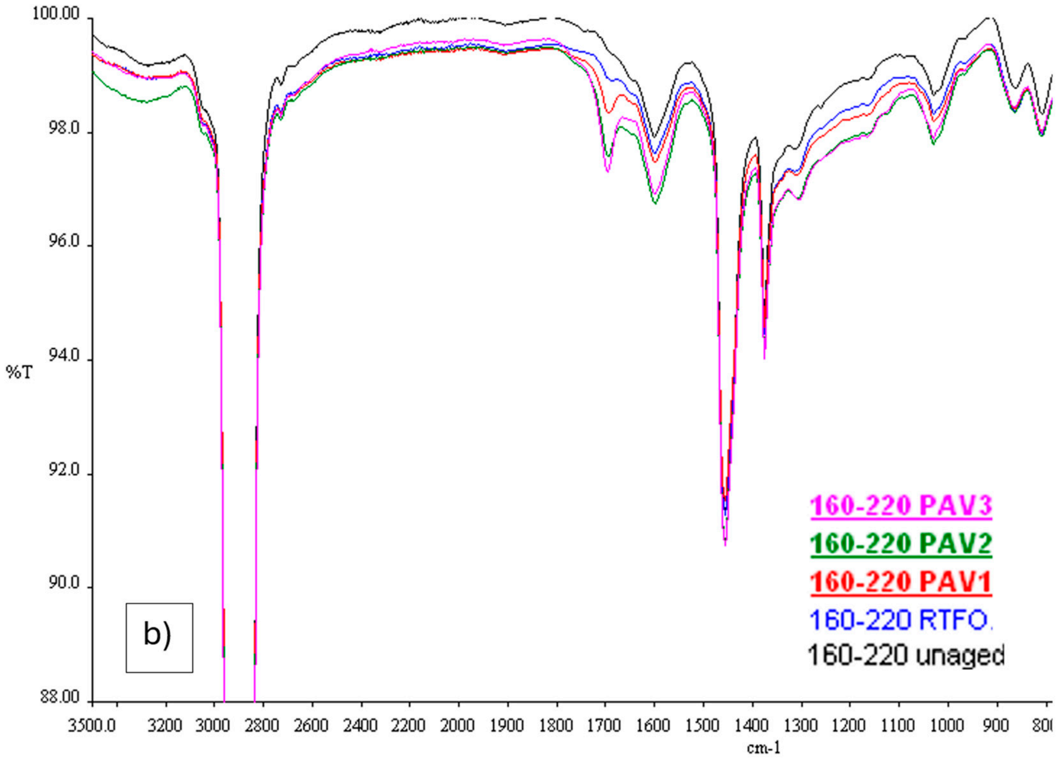

Figure S7. Representative ATR-FTIR spectra of the 160–220 asphalt binder at different aging stages, processed with ATR correction and automatic baseline correction: (a) full spectral range; (b) zoom of the diagnostic region used for the calculation of the carbonyl and sulfoxide indices. For clarity, one representative spectrum is shown for each aging condition.

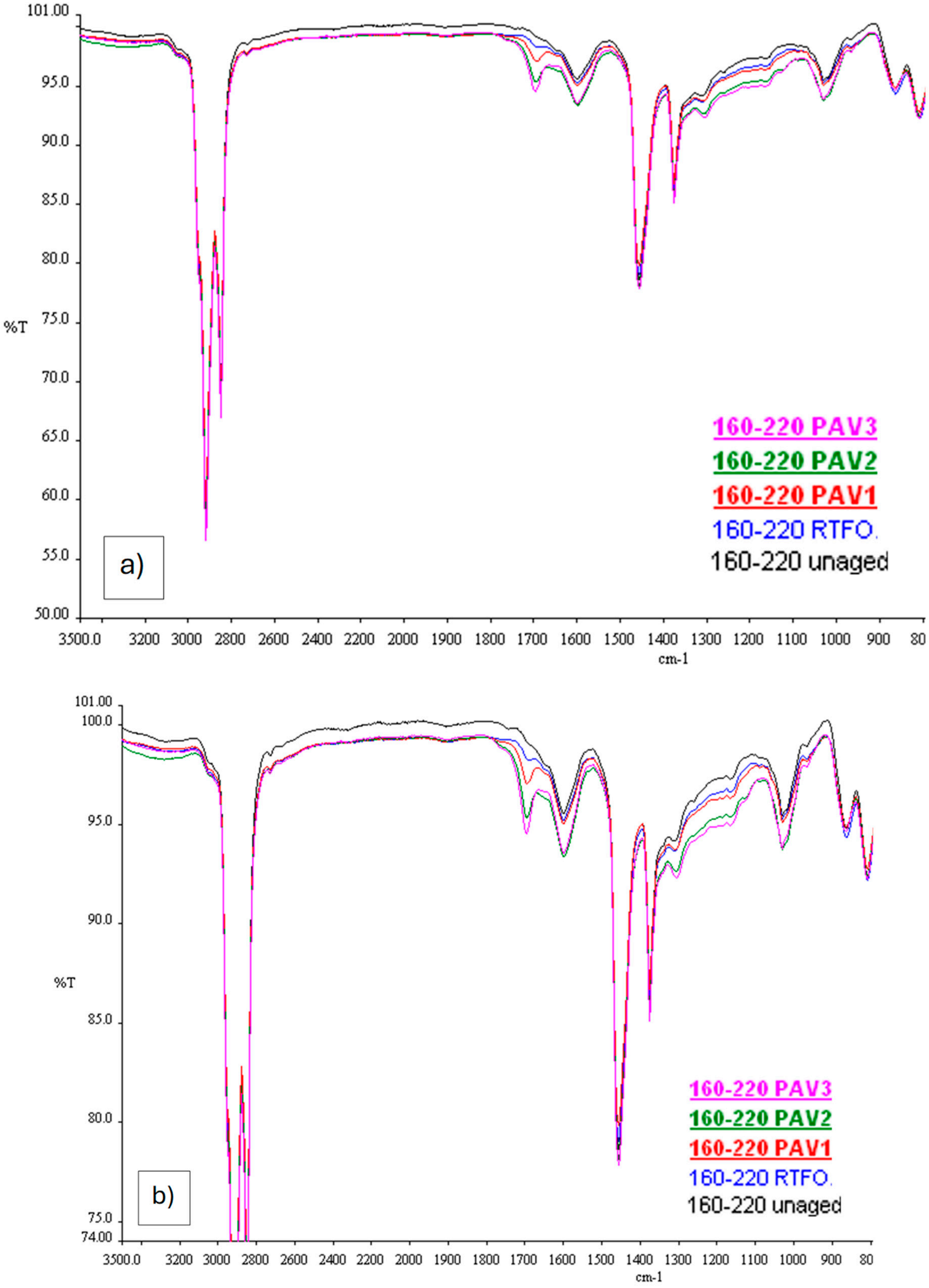

Figure S8. Representative ATR-FTIR spectra of the 160-220 asphalt binder at different aging stages, without ATR correction: (a) full spectral range; (b) zoom of the diagnostic region used for the calculation of the carbonyl and sulfoxide indices. For clarity, one representative spectrum is shown for each aging condition. All spectra were processed only by automatic baseline correction.

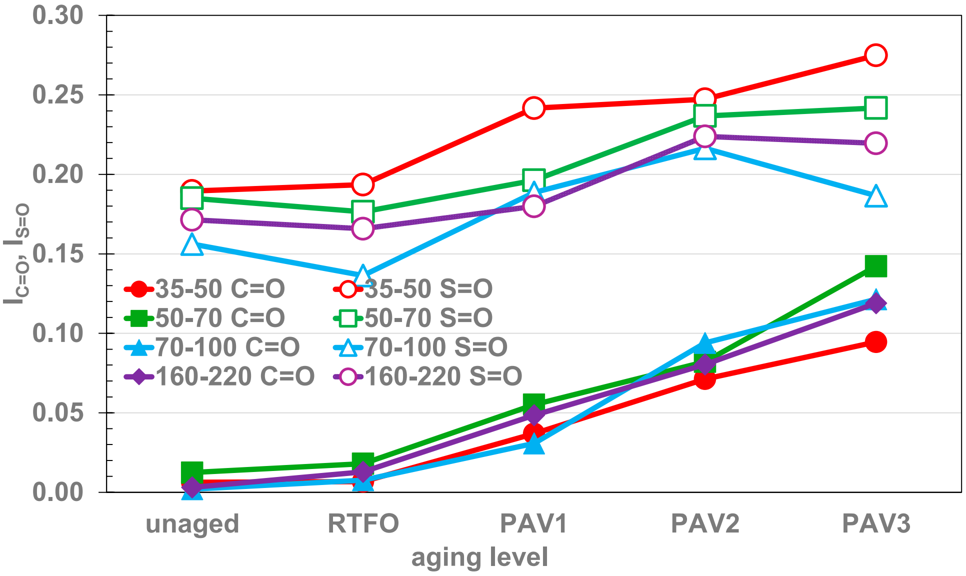

Figure S9. Carbonyl and sulfoxide indices calculated from ATR-FTIR spectra processed without ATR correction and using only automatic baseline correction for the investigated asphalt binders at different aging stages. Values were calculated from the average integrated areas obtained from three replicate FTIR spectra recorded for each sample.
